# Synthesis of pyrrolo[1,2-*a*]quinolines by formal 1,3-dipolar cycloaddition reactions of quinolinium salts

**DOI:** 10.3762/bjoc.15.149

**Published:** 2019-07-03

**Authors:** Anthony Choi, Rebecca M Morley, Iain Coldham

**Affiliations:** 1 Department of Chemistry, University of Sheffield, Brook Hill, Sheffield, S3 7HF, UK

**Keywords:** azomethine ylide, cycloaddition, heterocycle, pyrrolidine, stereoselective

## Abstract

Quinolinium salts, Q^+^-CH_2_-CO_2_Me Br^−^ and Q^+^-CH_2_-CONMe_2_ Br^−^ (where Q = quinoline), were prepared from quinolines. Deprotonation of these salts with triethylamine promoted the reaction of the resulting quinolinium ylides (formally azomethine ylides) with electron-poor alkenes by conjugate addition followed by cyclization or by [3 + 2] dipolar cycloaddition. The pyrroloquinoline products were formed as single regio- and stereoisomers. These could be converted to other derivatives by Suzuki–Miyaura coupling, reduction or oxidation reactions.

## Introduction

**Scheme 1:**
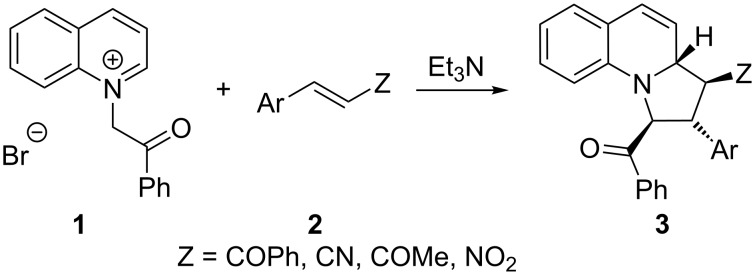
Reaction of ketone **1** with electron-deficient alkenes **2**.

Cycloaddition reactions of azomethine ylides are an important class of pericyclic reactions that give rise to pyrrolidine rings, prevalent in a large number of natural products and bioactive compounds. Many methods have been used to prepare azomethine ylides that undergo cycloaddition with π-systems, especially electron-poor alkenes to give pyrrolidine products [1–4]. Azomethine ylides can be classed either as stabilised or non-stabilised, depending on the presence or absence of an electron-withdrawing substituent such as a carbonyl group. The most common method for their preparation is by condensation of a secondary amine with an aldehyde to give an iminium ion that loses a proton to give the ylide, or by condensation of a primary amine with an aldehyde to give an imine followed by prototropy or deprotonation to give *N*-metalated azomethine ylides (see, for example, [5–18]). An alternative method is to prepare a salt of a heterocycle, typically by *N*-alkylation of a pyridine [19–27], isoquinoline [26–32], or related structures [33–35], followed by deprotonation. Such ylides are formally azomethine structures assuming reactivity of the aromatic ring as an iminium ion, although the reaction with electron-poor alkenes occurs through a stepwise conjugate addition–cyclization process [23]. We were interested in the related quinolinium ylides that, on (formal) cycloaddition would provide pyrrolo[1,2-*a*]quinolines as products. These are tricyclic compounds consisting of a pyrrole ring fused with a quinoline. Pyrroloquinolines have been found to show antibacterial and antifungal activity, to be ligands for the NK_1_ receptor, and to be effective against the Hif hypoxia pathway in cancer cell lines [36–38]. Almost all of the examples of dipolar cycloaddition reactions involving quinolinium salts that have been reported in the literature involve ketones as electron-withdrawing groups to stabilise the intermediate ylide [39–49]; for example, the ketone **1** is known to undergo reaction with alkenes **2** (Z = electron-withdrawing group) to give the tricyclic products **3** (Scheme 1) [41,49]. Similar examples with phenanthridinium and related ylides make use of ketones to stabilise the ylide [50–54]. The only exception (as far as we are aware) to the use of quinolinium ylides with ketones as stabilising groups are isolated reports with a carboxylic acid derivative, particular an ethyl ester group [55–59]. Here we describe a wider scope that extends the examples to alternative carbonyl derivatives and alternative alkenes, hence providing novel pyrroloquinoline compounds.

## Results and Discussion

**Scheme 2:**
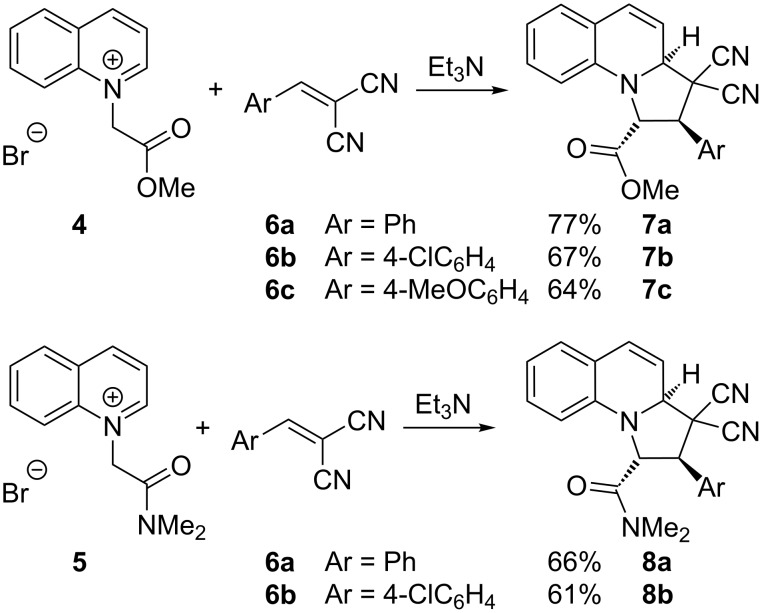
Reactions of ester **4** and amide **5** with electron-deficient alkenes **6**.

To test the feasibility of the reaction of quinolinium salts bearing electron-withdrawing groups other than ketones, we prepared ester **4** [55] and amide **5** by alkylation of quinoline. Arylidenemalononitriles such as **6a** are known to undergo related chemistry [41], so we heated this compound with the quinolinium salts in the presence of triethylamine and were pleased to obtain good yields of the adducts **7a**–**c** and **8a**,**b** (Scheme 2).

**Figure 1:**
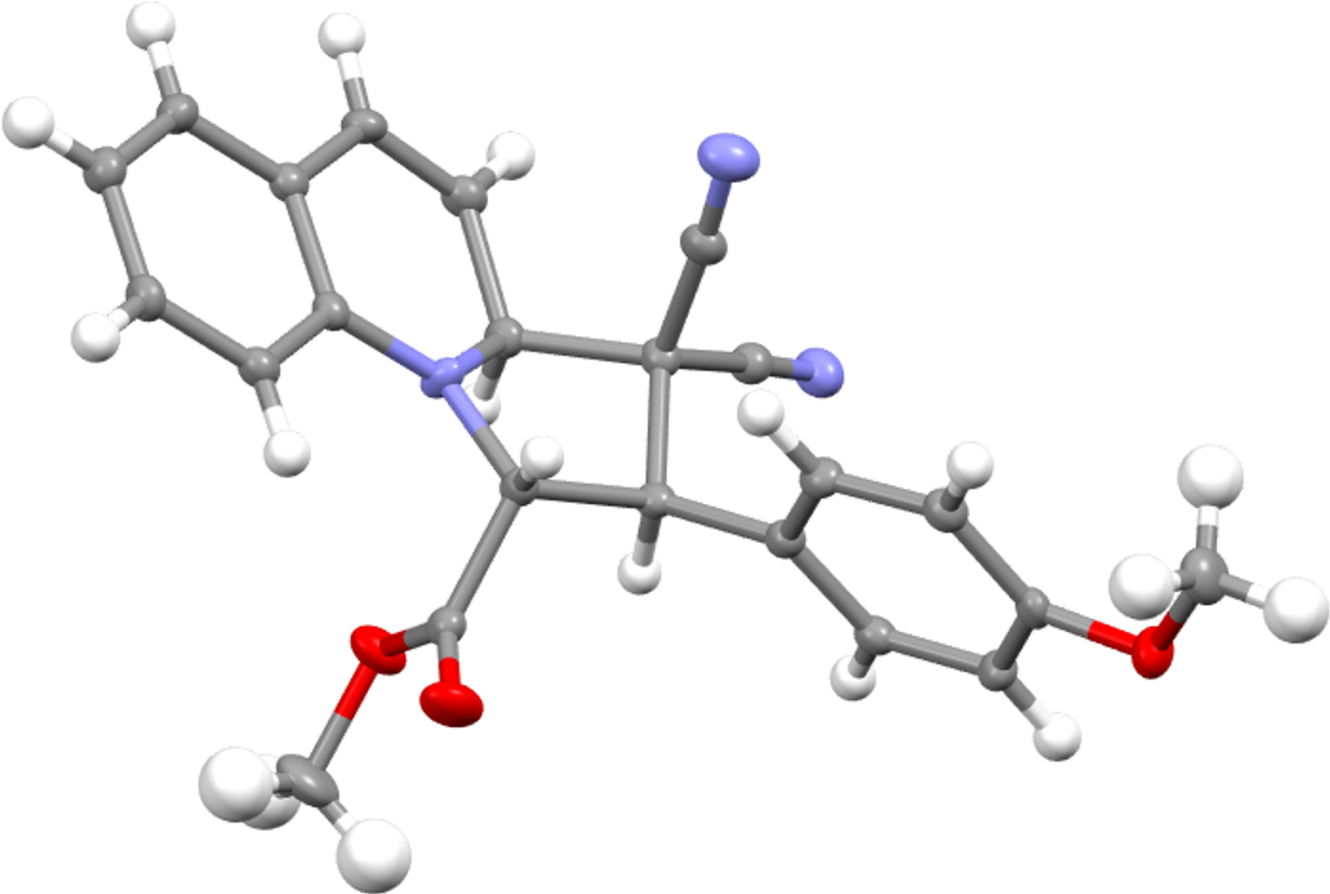
Single crystal X-ray structure for **7c**.

In each case, the products **7a**–**c** and **8a**,**b** were formed as a single regioisomer and stereoisomer. The selectivity in favour of the isomer drawn in Scheme 2 was verified by single crystal X-ray analysis of the adduct **7c** (Figure 1). The other isomers had similar coupling constants between adjacent protons and were assumed to have the same relative configuration.

**Scheme 3:**
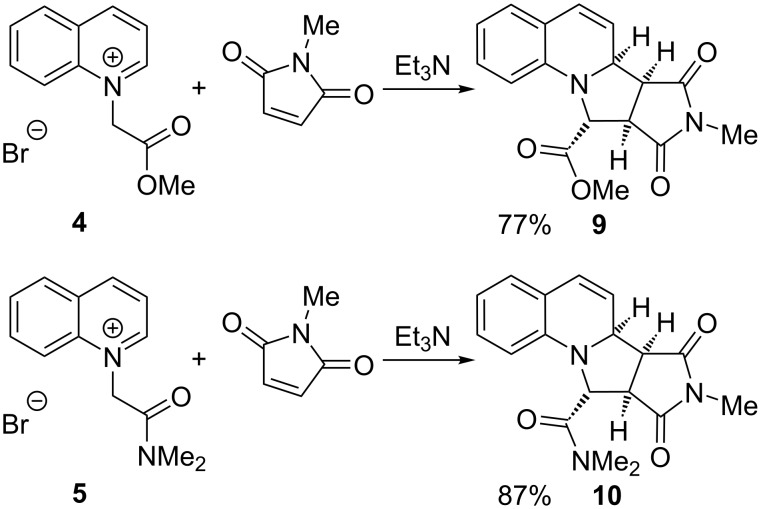
Reactions of ester **4** and amide **5** with *N*-methylmaleimide.

**Figure 2:**
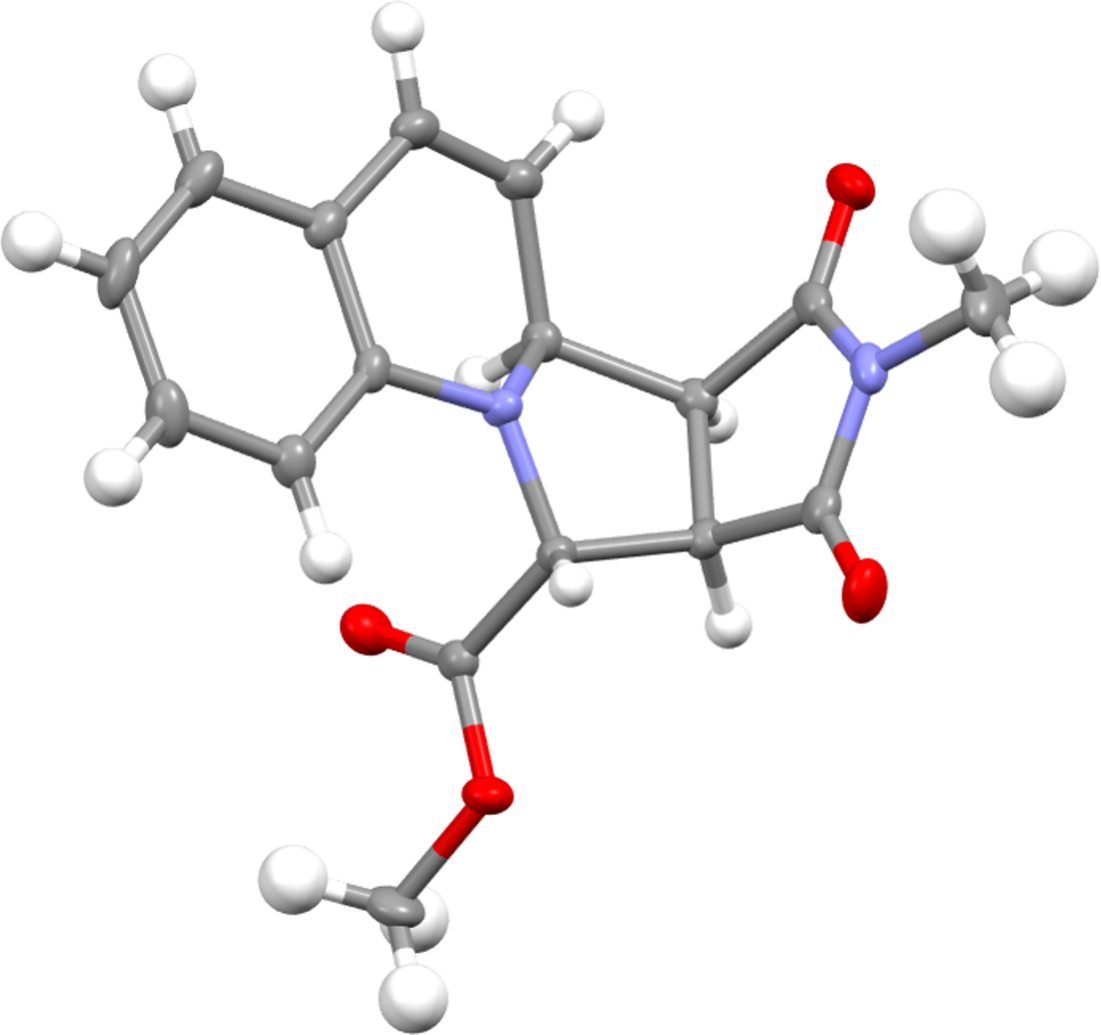
Single crystal X-ray structure for **9**.

A dipolarophile that has not been reported for reaction with quinolinium salts is *N*-methylmaleimide. We therefore tested the ability of this unsaturated compound to undergo reaction with the ylides derived from the salts **4** and **5**. In both cases, very good yields of the tetracyclic adducts **9** and **10** were obtained after heating for only 1 h (Scheme 3). The relative stereochemistry of the adduct **9** was determined by single crystal X-ray analysis (Figure 2).

**Scheme 4:**
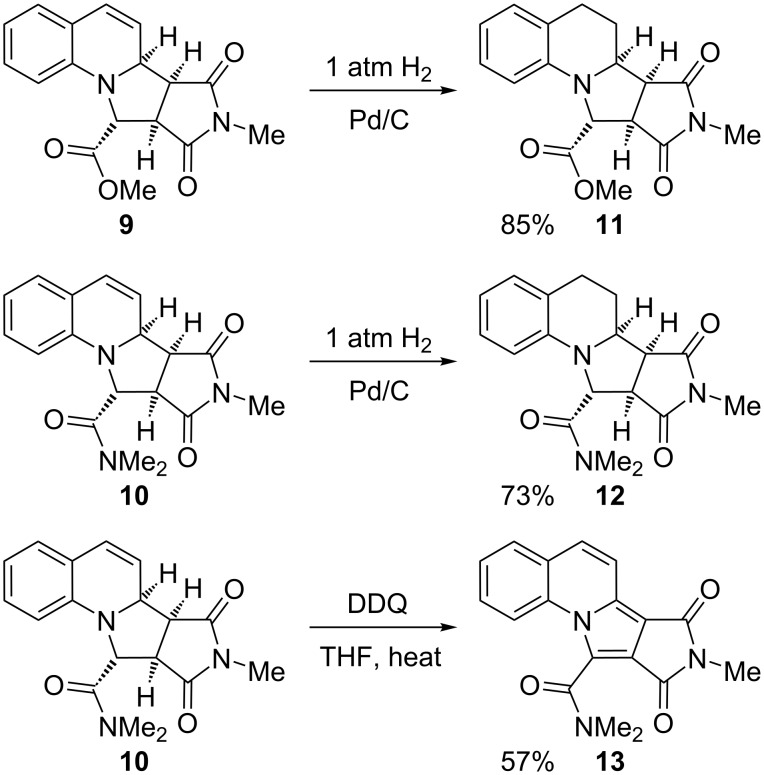
Reduction and oxidation of adducts **9** and **10**.

To explore the diversity of products that could be obtained from these adducts, we carried out a reduction of the alkene in compounds **9** and **10** by using hydrogen and palladium on charcoal (Scheme 4). This provides the tetrahydroquinoline adducts **11** and **12**. Additionally, and in contrast, oxidation of the adduct **10** was performed using the oxidant 2,3-dichloro-5,6-dicyanoquinone (DDQ) to give the fully unsaturated product **13**.

**Scheme 5:**
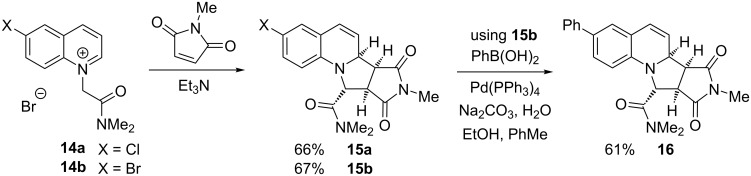
Formation of amides **15a** and **15b** and Suzuki–Miyaura coupling to yield **16**.

To expand the range of products and explore the scope of the reaction further, we prepared the salts **14a** and **14b** (from 6-chloroquinoline and 6-bromoquinoline) and these were heated with *N*-methylmaleimide in the presence of triethylamine in methanol to give the desired adducts **15a** and **15b** as single stereoisomers (Scheme 5). The stereochemistry of product **15a** was confirmed by single crystal X-ray analysis (see Supporting Information File 1) and matches the relative configuration of the adducts **9** and **10**. The bromide **15b** was coupled with phenylboronic acid using palladium catalysis to give derivative **16** (the chloride **15a** was inert under these conditions). The ability to prepare halogenated derivatives and to undergo palladium coupling demonstrates further versatility of these types of products.

## Conclusion

In conclusion, we have found that carboxylic ester and amide-stabilised anions derived from quinolinium salts react with arylidenemalononitriles and *N*-methylmaleimide to give adducts in good yields and with very high stereoselectivity (single isomer products were isolated). The adducts could be reduced, oxidised, or could undergo Suzuki–Miyaura coupling to give different substituted dihydro- and tetrahydroquinoline derivatives.

## Supporting Information

File 1 Experimental procedures, spectroscopic and X-ray data (CCDC 1907018–1907020 for compounds **7c**, **9** and **15a**) and copies of NMR spectra.
