# Supplementary material for: Synthesis of pyrrolo[1,2-a]quinolines by formal 1,3-dipolar cycloaddition reactions of quinolinium salts
Source: Beilstein J Org Chem. 2019 Jul 3;15:1480–4. doi: 10.3762/bjoc.15.149 (PMC6633155; doi:10.3762/bjoc.15.149)

BEILSTEIN JOURNAL OF ORGANIC CHEMISTRY

Supporting Information

for

Synthesis of pyrrolo[1,2-*a*]quinolines by formal 1,3-dipolar cycloaddition reactions of quinolinium salts

Anthony Choi, Rebecca M. Morley and Iain Coldham

Beilstein J. Org. Chem. **2019,** *15,* 1480–1484. doi:10.3762/bjoc.15.149

Experimental procedures, spectroscopic and X-ray data (CCDC 1907018–1907020 for compounds 7c, 9 and 15a) and copies of NMR spectra

License and Terms: This is a supporting information file under the terms of the Creative Commons Attribution License (http://creativecommons.org/licenses/by/4.0). Please note that the reuse, redistribution and reproduction in particular requires that the authors and source are credited.
The license is subject to the *Beilstein Journal of Organic Chemistry* terms and conditions: (https://www.beilstein-journals.org/bjoc)

Contents

1. General experimental details S2

2. Experimental procedures and characterisation data S3

3. Single crystal X-ray data

   3.1 Data for ester **7c** S18

   3.2 Data for ester **9** S25

   3.3 Data for amide **15a** S31

4. References S37

5. NMR spectra S38

1. General experimental details

All reagents were obtained from commercial suppliers and were used without further purification unless otherwise specified. Solvents were purified using a Grubbs dry solvent system.

Thin layer chromatography (TLC) analysis was performed on Merck silica gel 60 F_{254} plates and visualised by UV irradiation at 254 nm or by staining with an alkaline $KMnO_4$ dip. Flash column chromatography was carried out on VWR silica gel (40–63 micron mesh). Petrol refers to petroleum ether, b.p. 40−60 °C. ^{1}H proton NMR spectra were recorded on a Bruker AC400 (400 MHz) instrument in deuterochloroform, hexadeuterodimethyl sulfoxide or deuterium oxide. All chemical shifts are expressed in parts-per-million (ppm) with respect to the residual solvent peaks. Coupling constants (*J*) are given in Hz to the nearest 0.5 Hz. The following abbreviations are used singularly or in combination to indicate the multiplicity of signals: s = singlet, d = doublet, t = triplet, q=quartet, quin = quintet, sep = septet, m = multiplet, br = broad. ^{13}C NMR were recorded on the same instrument above at 100 MHz. Low and high resolution (accurate mass) mass spectra were recorded on a Micromass Autospec for Electron Impact (EI) and on a Walter LCT instrument for electrospray (ES). Infrared (IR) spectra were recorded on a Perkin-Elmer Spectrum RX Fourier Transform IR System. Only selected peaks are reported and absorption maxima are given in cm^{-1}. Melting points were recorded using a Gallenkamp hot stage and were uncorrected.

For the single crystal X-ray analyses, a suitable crystal was selected and mounted on a Mitigen microloop in fomblyn oil on a Bruker APEX-II CCD diffractometer. The crystal was kept at 100 K during data collection. Using Olex2,[1] the structure was solved with the ShelXT[2] structure solution program using Intrinsic Phasing and refined with the ShelXL[3] refinement package using Least Squares minimisation.

2. Experimental procedures and characterisation data

1-(2-Methoxy-2-oxoethyl)quinolin-1-ium bromide (4)

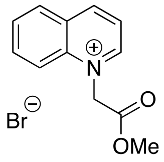

4

To a stirred solution of quinoline (4.57 mL, 38.7 mmol) in PhMe (50 mL) was added methyl bromoacetate (4.02 mL, 42.6 mmol). The mixture was heated at 65 °C for 16 h and was then cooled to room temperature. The mixture was filtered, was washed with Et_2O (200 mL) and was dried in air for 30 min to give ester **4** (4.7 g, 43%) as an orange amorphous solid; m.p. 149–151 °C (lit.[4] 152–153 °C); ^{1}H NMR (400 MHz, D_2O) δ = 9.13–9.09 (2H, m, 2 × CH), 8.26 (1H, d, *J* = 8.5 Hz, CH), 8.13–8.08 (2H, m, 2 × CH), 7.97 (1H, dd, *J* = 8.5, 6.0 Hz, CH), 7.91–7.87 (1H, m, CH), 5.89 (2H, s, CH_2), 3.72 (3H, s, CH_3); ^{13}C NMR (100 MHz, D_2O) δ = 167.7 (C=O), 150.2 (CH), 149.6 (CH), 138.6 (C), 136.7 (CH), 131.0 (CH), 130.3 (CH), 130.0 (C), 121.7 (CH), 117.7 (CH), 57.7 (CH_2), 53.9 (CH_3). Data consistent with the literature.[4]

1-[(Dimethylcarbamoyl)methyl]quinolin-1-ium bromide (5)

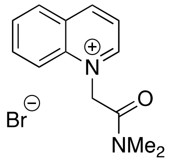

5

To a stirred solution of quinoline (1.8 mL, 15.5 mmol) in PhMe (20 mL) was added 2-bromo-*N*,*N*-dimethylacetamide[5] (2.8 g, 17 mmol). The mixture was heated at 65 °C for 16 h and was then cooled to room temperature. The mixture was filtered, was washed with Et_2O (200 mL) and was dried in air for 30 min to give amide **5** (2.2 g, 47%) as an off-white amorphous solid; m.p. 175–177 °C; FT-IR ν_{max} (film)/cm^{-1} 3407, 2911, 1653 (C=O), 1586, 1528, 1405, 1369, 1147, 804, 771, 597, 507; ^{1}H NMR (400 MHz, D_2O) δ = 9.11–9.03 (2H, m, 2 × CH), 8.26 (1H, dd, *J* = 8.0, 1.0 Hz, CH), 8.10–7.86 (4H, m, 4 × CH), 6.02 (2H, s, CH_2), 3.18 (3H, s, CH_3), 2.89 (3H, s, CH_3); ^{13}C NMR (100 MHz, D_2O) δ = 165.5

(C=O), 150.0 (CH), 149.2 (CH), 138.9 (C), 136.4 (CH), 130.8 (CH), 130.2 (CH), 129.9 (C), 121.6 (CH), 117.9 (CH), 58.4 (CH_2), 36.4 (CH_3), 35.9 (CH_3); HRMS *m/z* (ES) Found: M^+, 215.1181. $C_{13}H_{15}N_2O$ requires M^+ 215.1179; LRMS *m/z* (ES) 215 (100%, M^+).

Methyl 3,3-dicyano-2-phenyl-1*H*,2*H*,3*H*,3a*H*-pyrrolo[1,2-*a*]quinoline-1-carboxylate (7a)

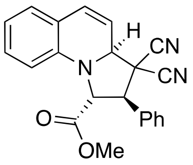

7a

To a stirred suspension of ester **4** (282 mg, 1 mmol) in MeOH (6 mL) was added arylidenemalononitrile **6a**[6] (154 mg, 1 mmol) followed by Et_3N (0.14 mL, 1 mmol). The mixture was heated under reflux for 1 h and was then cooled to room temperature. The solvent was removed under reduced pressure and the crude product was purified by column chromatography on silica gel, eluting with petrol–EtOAc (9:1), to give ester **7a** (274 mg, 77%) as a yellow amorphous solid; m.p. 111–113 °C; R_f 0.63 [petrol–EtOAc (1:1)]; FT-IR ν_{max} (film)/cm^{-1} 3200, 2952, 2491, 2154 (C≡N), 1735 (C=O), 1647, 1594, 1451, 1203, 727, 701; ^{1}H NMR (400 MHz, $CDCl_3$) δ = 7.57–7.49 (5H, m, 5 × CH), 7.16 (1H, td, *J* = 7.5, 1.5 Hz, CH), 7.02 (1H, dd, *J* = 7.5, 1.5 Hz, CH), 6.82–6.74 (2H, m, 2 × CH), 6.42 (1H, dd, *J* = 8.0, 2.0 Hz, CH), 5.82 (1H, dd, *J* = 8.0, 2.5 Hz, CH), 5.66 (1H, dd, *J* = 2.5, 2.0 Hz, CH), 4.77 (1H, d, *J* = 8.5 Hz, CH), 4.08 (1H, d, *J* = 8.5 Hz, CH), 3.81 (3H, s, CH_3); ^{13}C NMR (100 MHz, $CDCl_3$) δ = 171.6 (C=O), 141.1 (C), 131.2 (C), 131.2 (CH), 130.5 (CH), 130.1 (CH), 129.5 (CH), 128.4 (CH), 128.4 (CH), 119.8 (CH), 119.3 (C), 114.9 (CH), 112.6 (CN), 111.4 (CN), 109.8 (CH), 69.9 (CH), 64.6 (CH), 56.3 (CH), 53.2 (CH_3), 48.6 (C); HRMS *m/z* (ES) Found: MH^+, 356.1399. $C_{22}H_{18}N_3O_2$ requires MH^+ 356.1394; LRMS *m/z* (ES) 356.1 (100%, MH^+).

Methyl 2-(4-chlorophenyl)-3,3-dicyano-1*H*,2*H*,3*H*,3a*H*-pyrrolo[1,2-*a*]quinoline-1-carboxylate (7b)

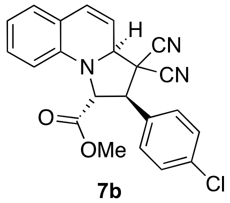

7b

To a stirred suspension of ester **4** (282 mg, 1 mmol) in MeOH (6 mL) was added arylidenemalononitrile **6b**[6] (189 mg, 1 mmol) followed by Et_3N (0.14 mL, 1 mmol). The mixture was heated under reflux for 1 h and was then cooled to room temperature. The solvent was removed under reduced pressure and the crude product was purified by column chromatography on silica gel, eluting with petrol–EtOAc (9:1), to give ester **7b** (260 mg, 67%) as a yellow amorphous solid; m.p. 95–98 °C; R_f 0.67 [petrol–EtOAc (1:1)]; FT-IR ν_{max} (film)/cm^{-1} 2954, 2489, 2162 (C≡N), 1740 (C=O), 1648, 1594, 1453, 1254, 1198, 735, 699; ^{1}H NMR (400 MHz, $CDCl_3$) δ = 7.48 (4H, m, 4 × CH), 7.16 (1H, td, *J* = 7.5, 1.5 Hz, CH), 7.02 (1H, dd, *J* = 7.5, 1.5 Hz, CH), 6.82–6.74 (2H, m, 2 × CH), 6.40 (1H, dd, *J* = 10.0, 1.5 Hz, CH), 5.81 (1H, dd, *J* = 10.0, 2.5 Hz, CH), 5.64 (1H, dd, *J* = 2.5, 1.5 Hz, CH), 4.70 (1H, d, *J* = 8.5 Hz, CH), 4.05 (1H, d, *J* = 8.5 Hz, CH), 3.81 (3H, s, CH_3); ^{13}C NMR (100 MHz, $CDCl_3$) δ = 171.4 (C=O), 141.0 (C), 136.3 (C), 131.3 (CH), 130.6 (CH), 129.8 (2 x CH), 129.7 (C), 128.5 (CH), 119.9 (CH), 119.3 (C), 114.8 (CH), 112.4 (CN), 111.3 (CN), 109.8 (CH), 69.9 (CH), 64.6 (CH), 55.7 (CH), 53.3 (CH_3), 48.5 (C); HRMS *m/z* (ES) Found: MH^+, 390.1007. $C_{22}H_{17}N_3O_2{}^{35}Cl$ requires MH^+ 390.1004; MH^+, 392.0989. $C_{22}H_{17}N_3O_2{}^{37}Cl$ requires MH^+ 392.0974; LRMS *m/z* (ES) 390.1 (100%, MH^+ for ^{35}Cl), 392.1 (35%, MH^+ for ^{37}Cl).

Methyl 3,3-dicyano-2-(4-methoxyphenyl)-1*H*,2*H*,3*H*,3a*H*-pyrrolo[1,2-*a*]quinoline-1-carboxylate (7c)

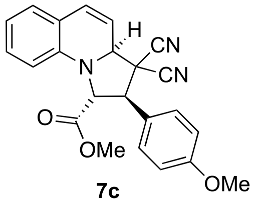

7c

To a stirred suspension of ester **4** (282 mg, 1 mmol) in MeOH (6 mL) was added arylidenemalononitrile **6c**[6] (184 mg, 1 mmol) followed by Et_3N (0.14 mL, 1 mmol). The mixture was heated under reflux for 1 h and was then cooled to room temperature. The solvent was removed under reduced pressure and the crude product was purified by column chromatography on silica gel, eluting with petrol–EtOAc (9:1), to give ester **7c** (245 mg, 64%) as yellow needles; m.p. 130–133 °C; R_f 0.59 [petrol–EtOAc (1:1)]; FT-IR ν_{max} (film)/cm^{-1} 2944, 2221, 1737 (C=O), 1604 (C=C), 1571, 1510, 1257, 1177, 1021, 835, 755, 569, 525; ^{1}H NMR (400 MHz, DMSO-d_6) δ = 7.57–7.54 (2H, m, 2 × CH), 7.21–7.19 (1H, m, CH), 7.07–7.03 (3H, m, 3 × CH), 6.79 (1H, dd, *J* = 10.0, 2.0 Hz, CH), 6.73–6.69 (1H, m, CH), 6.39 (1H, d, *J* = 8.0 Hz, CH), 5.89 (1H, dd, *J* = 10.0, 2.5 Hz, CH), 5.62 (1H, dd, *J* = 2.5, 2.0 Hz, CH), 4.91 (1H, d, *J* = 8.5 Hz, CH), 4.57 (1H, d, *J* = 8.5 Hz, CH), 3.80 (3H, s, CH_3), 3.67 (3H, s, CH_3); ^{13}C NMR (100 MHz, DMSO-d_6, one CH missing/overlapping) δ = 171.0 (C=O), 160.4 (C), 141.5 (C), 130.7 (CH), 130.4 (CH), 128.2 (CH), 124.7 (C), 119.2 (C), 119.1 (CH), 116.3 (CH), 114.9 (CH), 112.9 (2 × CN), 110.7 (CH), 68.5 (CH), 63.0 (CH), 55.7 (CH), 53.6 (CH_3), 53.2 (CH_3), 49.3 (C); HRMS *m/z* (ES) Found: MH^+, 386.1502. $C_{23}H_{20}N_3O_3$ requires MH^+ 386.1499; LRMS *m/z* (ES) 386.2 (100%, MH^+). CCDC 1907018.

3,3-Dicyano-*N,N*-dimethyl-2-phenyl-1*H*,2*H*,3*H*,3a*H*-pyrrolo[1,2-*a*]quinoline-1-carboxamide (8a)

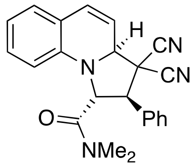

8a

To a stirred suspension of amide **5** (295 mg, 1 mmol) in MeOH (6 mL) was added arylidenemalononitrile **6a**[6] (154 mg, 1 mmol) followed by Et_3N (0.14 mL, 1 mmol). The mixture was heated under reflux for 1 h and was then cooled to room temperature. The solvent was removed under reduced pressure and the crude product was purified by column chromatography on silica gel, eluting with petrol–EtOAc (9:1), to give amide **8a** (243 mg, 66%) as a light brown amorphous solid; m.p. 87–90 °C; R_f 0.28 [petrol–EtOAc (1:1)]; FT-IR ν_{max} (film)/cm^{-1} 3034, 2492, 2162 (C≡N), 1650 (C=O), 1571, 1597, 1489, 1464, 1400, 1374, 1236, 1134, 740, 699, 625; ^{1}H NMR (400 MHz, DMSO-d_6) δ = 7.78–7.72 (2H, m, 2 × CH), 7.53–7.45 (3H, m, 3 × CH), 7.11–7.03 (2H, m, 2 × CH), 6.77 (1H, dd, *J* = 10.0, 2.0 Hz, CH), 6.68 (1H, td, *J* = 7.5, 0.5 Hz, CH), 6.14 (1H, d, *J* = 7.5 Hz, CH), 5.91 (1H, dd, *J* = 10.0, 2.0 Hz, CH), 5.71 (1H, t, *J* = 2.0 Hz, CH), 5.33 (1H, d, *J* = 7.5 Hz, CH), 4.29 (1H, d, *J* = 7.5 Hz, CH), 2.83 (3H, s, CH_3), 2.69 (3H, s, CH_3); ^{13}C NMR (100 MHz, DMSO-d_6) δ = 169.8 (C=O), 141.5 (C), 133.1 (C), 130.5 (CH), 130.5 (CH), 130.1 (CH), 129.6 (CH), 129.6 (CH), 128.1 (CH), 119.8 (C), 118.7 (CH), 116.9 (CH), 113.2 (2 × CN), 110.5 (CH), 69.7 (CH), 59.0 (CH), 55.7 (CH), 48.7 (C), 37.0 (CH_3), 36.1 (CH_3); HRMS *m/z* (ES) Found: MH^+, 369.1715. $C_{23}H_{21}N_4O$ requires MH^+ 369.1710; LRMS *m/z* (ES) 369.2 (100%, MH^+).

2-(4-Chlorophenyl)-3,3-dicyano-*N,N*-dimethyl-1*H*,2*H*,3*H*,3a*H*-pyrrolo[1,2-*a*]quinoline-1-carboxamide (8b)

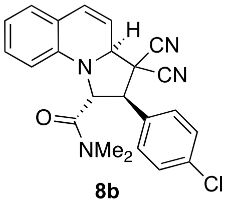

8b

To a stirred suspension of amide **5** (295 mg, 1 mmol) in MeOH (6 mL) was added arylidenemalononitrile **6b**[6] (189 mg, 1 mmol) followed by Et_3N (0.14 mL, 1 mmol). The mixture was heated under reflux for 1 h and was then cooled to room temperature. The solvent was removed under reduced pressure and the crude product was purified by column chromatography on silica gel, eluting with petrol–EtOAc (9:1), to give amide **8b** (245 mg, 61%) as a light brown amorphous solid; m.p. 120–123 °C; R_f 0.51 [petrol–EtOAc (1:1)]; FT-IR ν_{max} (film)/cm^{-1} 3054, 2936, 2489, 2162, 2026, 1645 (C=O), 1492, 1459, 1402, 1374, 1136, 1088, 1011, 830, 778, 742, 622, 507; ^{1}H NMR (400 MHz, DMSO-d_6) δ = 7.83–7.80 (2H, m, 2 × CH), 7.61–7.57 (2H, m, 2 × CH), 7.11–7.03 (2H, m, 2 × CH), 6.76 (1H, dd, *J* = 10.0, 2.0 Hz, CH), 6.68 (1H, td, *J* = 7.5, 1.0 Hz, CH), 6.13 (1H, d, *J* = 8.0 Hz, CH), 5.91 (1H, dd, *J* = 10.0, 2.0 Hz, CH), 5.70 (1H, t, *J* = 2.0 Hz, CH), 5.34 (1H, d, *J* = 7.5 Hz, CH), 4.41 (1H, d, *J* = 7.5 Hz, CH), 2.84 (3H, s, CH_3), 2.71 (3H, s, CH_3); ^{13}C NMR (100 MHz, DMSO-d_6) δ = 169.6 (C=O), 141.4 (C), 134.9 (C), 132.3 (C), 131.5 (CH), 130.6 (CH), 130.5 (CH), 129.6 (CH), 128.0 (CH), 119.8 (2 x CN), 118.7 (CH), 116.9 (CH), 113.1 (C), 110.6 (CH), 69.7 (CH), 58.9 (CH), 54.7 (CH), 48.6 (C), 37.1 (CH_3), 36.1 (CH_3); HRMS *m/z* (ES) Found: MH^+, 403.1322. $C_{23}H_{20}N_4O^{35}Cl$ requires MH^+ 403.1326; MH^+, 405.1310. $C_{23}H_{20}N_4O^{37}Cl$ requires MH^+ 405.1291; LRMS *m/z* (ES) 403.1 (100%, MH^+ for ^{35}Cl), 405.1 (35%, MH^+ for ^{37}Cl).

Methyl 13-methyl-12,14-dioxo-1,13-diazatetracyclo[8.6.0.0^{2,7}.0^{11,15}]hexadeca-2(7),3,5,8-tetraene-16-carboxylate (9)

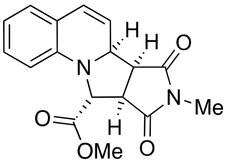

9

To a stirred suspension of ester **4** (141 mg, 0.5 mmol) in MeOH (3 mL) was added *N*-methylmaleimide (56 mg, 0.5 mmol) followed by Et_3N (0.07 mL, 0.5 mmol). The mixture was heated under reflux for 1 h and was then cooled to room temperature. The solvent was removed under reduced pressure and the crude product was purified by column chromatography on silica gel, eluting with petrol–EtOAc (3:1), to give ester **9** (120 mg, 77%) as pale yellow needles; m.p. 182–185 °C; R_f 0.26 [petrol–EtOAc (1:1)]; FT-IR ν_{max} (film)/cm^{-1} 2934, 1701 (C=O), 1638 (C=O), 1494, 1430, 1374, 1351, 1290, 1136, 1016, 753, 622; ^{1}H NMR (400 MHz, $CDCl_3$) δ = 7.09 (1H, td, *J* = 7.5, 1.5 Hz, CH), 6.96 (1H, dd, *J* = 7.5, 1.5 Hz, CH), 6.73 (1H, td, *J* = 7.5, 1.0 Hz, CH), 6.48 (1H, brd, *J* = 8.0 Hz, CH), 6.39 (1H, dd, *J* = 10.0, 2.0 Hz, CH), 6.07 (1H, dd, *J* = 10.0, 2.0 Hz, CH), 5.13 (1H, dt, *J* = 7.5, 2.0 Hz, CH), 4.82 (1H, s, CH), 3.82 (3H, s, CH_3), 3.59–3.52 (2H, m, 2 × CH), 2.98 (3H, s, CH_3); ^{13}C NMR (100 MHz, $CDCl_3$) δ = 176.6 (C=O), 175.8 (C=O), 170.5 (C=O), 141.1 (C), 129.2 (CH), 127.4 (CH), 126.5 (CH), 121.9 (C), 120.9 (CH), 119.0 (CH), 110.9 (CH), 60.8 (CH), 59.7 (CH), 52.7 (CH_3), 47.7 (CH), 47.0 (CH), 25.5 (CH_3); HRMS *m/z* (ES) Found: MH^+, 313.1188. $C_{17}H_{17}N_2O_4$ requires MH^+ 313.1183; LRMS *m/z* (ES) 313 (100%, MH^+), 335 (15%, MNa^+); Found: C, 65.04; H, 5.24; N, 8.58. $C_{17}H_{16}N_2O_4$ requires C, 65.38; H, 5.16; N, 8.97. CCDC 1907019.

_N_,_N_,13-Trimethyl-12,14-dioxo-1,13-diazatetracyclo[8.6.0.0^{2,7}.0^{11,15}]hexadeca-2(7),3,5,8-tetraene-16-carboxamide (10)

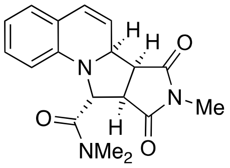

10

To a stirred suspension of amide **5** (295 mg, 1 mmol) in MeOH (6 mL) was added *N*-methylmaleimide (111 mg, 1 mmol) followed by Et_3N (0.14 mL, 1 mmol). The mixture was heated under reflux for 1 h and was then cooled to room temperature. The solvent was removed under reduced pressure and the crude product was purified by column chromatography on silica gel, eluting with petrol–EtOAc (1:1), to give amide **10** (282 mg, 87%) as an off-white amorphous solid; m.p. 155–158 °C; R_f 0.07 [petrol–EtOAc (1:1)]; FT-IR ν_{max} (film)/cm^{-1} 2671, 1696 (C=O), 1655 (C=O), 1489, 1436, 1290, 1139, 1116, 753, 612; ^{1}H NMR (400 MHz, $CDCl_3$) δ = 7.07–6.92 (2H, m, 2 × CH), 6.72–6.66 (1H, m, CH), 6.42–6.38 (1H, m, CH), 6.17–6.08 (2H, m, 2 × CH), 5.41 (1H, dt, *J* = 8.0, 2.0 Hz, CH), 4.96 (1H, s, CH), 3.52–3.49 (1H, m, CH), 3.42–3.39 (4H, m, CH & CH_3), 3.07 (3H, s, CH_3), 2.97 (3H, s, CH_3); ^{13}C NMR (100 MHz, $CDCl_3$) δ = 177.5 (C=O), 176.0 (C=O), 169.5 (C=O), 141.7 (C), 129.1 (CH), 127.4 (CH), 126.4 (CH), 122.4 (C), 121.4 (CH), 118.7 (CH), 110.1 (CH), 60.7 (CH), 59.3 (CH), 47.9 (CH), 47.5 (CH), 37.1 (CH_3), 35.9 (CH_3), 25.4 (CH_3); HRMS *m/z* (ES) Found: MH^+, 326.1506. $C_{18}H_{20}N_3O_3$ requires MH^+ 326.1499; LRMS *m/z* (ES) 229 (15%), 253 (5%), 326 (100%, MH^+).

Methyl 13-methyl-12,14-dioxo-1,13-diazatetracyclo[8.6.0.0^{2,7}.0^{11,15}]hexadeca-2(7),3,5-triene-16-carboxylate (11)

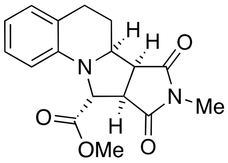

11

To a solution of ester **9** (100 mg, 0.32 mmol) in EtOAc (6 mL) was added 10% Pd/C (17 mg, 0.016 mmol). The mixture was stirred at room temperature under a hydrogen gas atmosphere (1 atm) for 48 h. The mixture was filtered through a pad of celite and the solvent was removed under reduced pressure. The crude product was purified using a silica gel plug, eluting with petrol–EtOAc (1:1), to give ester **11** (85 mg, 85%) as an off-white amorphous solid; m.p. 163–165 °C; R_f 0.26 [petrol–EtOAc (1:1)]; FT-IR ν_{max} (film)/cm^{-1} 2929, 1701 (C=O), 1640 (C=O), 1499, 1436, 1282, 1142, 1006, 755, 617, 587; ^{1}H NMR (400 MHz, $CDCl_3$) δ = 7.08–7.02 (2H, m, 2 × CH), 6.73 (1H, td, *J* = 7.5, 1.0 Hz, CH), 6.54 (1H, d, *J* = 7.5 Hz, CH), 4.91 (1H, s, CH), 4.17–4.09 (1H, m, CH), 3.77 (3H, s, CH_3), 3.53–3.49 (2H, m, 2 × CH), 3.05–2.96 (4H, m, CH & CH_3), 2.88–2.81 (1H, m, CH), 2.47–2.41 (1H, m, CH), 1.63–1.52 (1H, m, CH); ^{13}C NMR (100 MHz, $CDCl_3$) δ = 176.8 (C=O), 175.6 (C=O), 171.2 (C=O), 142.3 (C), 129.3 (CH), 127.1 (CH), 122.5 (C), 118.7 (CH), 112.2 (CH), 61.0 (CH), 58.0 (CH), 52.4 (CH_3), 48.6 (CH), 47.2 (CH), 27.6 (CH_2), 25.3 (CH_3), 25.2 (CH_2); HRMS *m/z* (ES) Found: MH^+, 315.1344. $C_{17}H_{19}N_2O_4$ requires MH^+ 315.1339; LRMS *m/z* (ES) 315 (100%, MH^+), 337 (15%, MNa^+).

_N_,_N_,13-Trimethyl-12,14-dioxo-1,13-diazatetracyclo[8.6.0.0^{2,7}.0^{11,15}]hexadeca-2(7),3,5-triene-16-carboxamide (12)

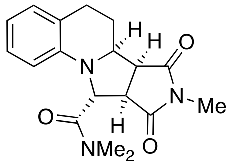

12

To a solution of amide **10** (100 mg, 0.31 mmol) in EtOAc (6 mL) was added 10% Pd/C (17 mg, 0.016 mmol). The mixture was stirred at room temperature under a hydrogen gas atmosphere (1 atm) for 48 h. The mixture was filtered through a pad of celite and the solvent was removed under reduced pressure. The crude product was purified using a silica gel plug, eluting with EtOAc, to give amide **12** (73 mg, 73%) as a brown amorphous solid; m.p. 195–197 °C; R_f 0.07 [petrol–EtOAc (1:1)]; FT-IR ν_{max} (film)/cm^{-1} 2929, 1701 (C=O), 1640 (C=O), 1499, 1436, 1282, 1142, 1006, 755, 617, 587; ^{1}H NMR (400 MHz, $CDCl_3$) δ = 7.05–6.99 (2H, m, 2 × CH), 6.68 (1H, t, *J* = 7.5 Hz, CH), 6.21 (1H, d, *J* = 7.5 Hz, CH), 5.10 (1H, s, CH), 4.49–4.43 (1H, m, CH), 3.51 (1H, t, *J* = 8.5 Hz, CH), 3.42 (3H, s, CH_3), 3.34 (1H, d, *J* = 8.5 Hz, CH), 3.11–2.97 (7H, m, CH & 2 × CH_3), 2.88–2.80 (1H, m, CH), 2.47–2.40 (1H, m, CH), 1.66–1.55 (1H, m, CH); ^{13}C NMR (100 MHz, $CDCl_3$) δ = 177.7 (C=O), 175.9 (C=O), 170.5 (C=O), 142.9 (C), 129.4 (CH), 127.0 (CH), 122.4 (C), 118.3 (CH), 111.1 (CH), 59.0 (CH), 58.8 (CH), 48.8 (CH), 47.5 (CH), 37.0 (CH_3), 35.8 (CH_3), 27.7 (CH_2), 25.4 (CH_2), 25.2 (CH_3); HRMS *m/z* (ES) Found: MH^+, 328.1661. $C_{18}H_{22}N_3O_3$ requires MH^+ 328.1656; LRMS *m/z* (ES) 328 (100%, MH^+).

_N_,_N_,13-Trimethyl-12,14-dioxo-1,13-diazatetracyclo[8.6.0.0^{2,7}.0^{11,15}]hexadeca-2(7),3,5,8,10,15-hexaene-16-carboxamide (13)

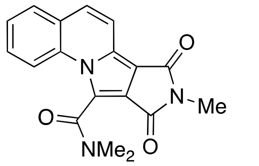

13

To a suspension of amide **10** (163 mg, 0.5 mmol) in dry THF (2 mL) was added 2,3-dichloro-5,6-dicyano-*p*-benzoquinone (227 mg, 1 mmol). The mixture was heated under reflux for 10 minutes then was cooled to room temperature. The solvent was removed under reduced pressure and the mixture was diluted with CH_2Cl_2 (100 mL) and filtered. The solvent was evaporated and the crude product was purified by column chromatography on silica gel, eluting with petrol–EtOAc (3:2), to give amide **13** (92 mg, 57%) as a yellow amorphous solid; m.p. 262–264 °C; R_f 0.40 [petrol–EtOAc (1:4)]; FT-IR ν_{max} (film)/cm^{-1} 3054, 2933, 1752, 1693 (C=O), 1632 (C=O), 1567, 1427, 1392, 1348, 979, 958, 818, 757, 736; ^{1}H NMR (400 MHz, $CDCl_3$) δ = 7.87 (1H, d, *J* = 8.5 Hz, CH), 7.76 (1H, d, *J* = 8.0 Hz, CH), 7.66 (1H, d, *J* = 9.5 Hz, CH), 7.60 (1H, t, *J* = 8.0 Hz, CH), 7.54–7.46 (2H, m, 2 × CH), 3.34 (3H, s, CH_3), 3.29 (3H, s, CH_3), 3.12 (3H, s, CH_3); ^{13}C NMR (100 MHz, $CDCl_3$) δ = 164.2 (C=O), 164.0 (C=O), 162.1 (C=O), 133.6 (C), 129.8 (CH), 129.6 (C), 129.5 (CH), 127.8 (CH), 126.0 (CH), 125.0 (C), 124.4 (C), 118.4 (C), 117.3 (CH), 116.8 (CH), 110.9 (C), 38.5 (CH_3), 35.5 (CH_3), 24.2 (CH_3); HRMS *m/z* (ES) Found: MH$^+$, 322.1186. $C_{18}H_{16}N_3O_3$ requires MH$^+$ 315.1186; LRMS *m/z* (ES) 201.2 (25%), 274.3 (40%), 322.1 (55%, MH$^+$), 344.1 (100%, MNa$^+$).

6-Chloro-1-[(dimethylcarbamoyl)methyl]quinolin-1-ium bromide (14a)

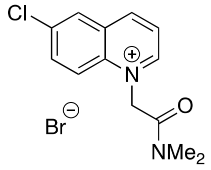

14a

To a solution of 6-chloroquinoline (714 mg, 4.36 mmol) in PhMe (6 mL) was added 2-bromo-*N,N*-dimethylacetamide[5] (0.52 mL, 4.8 mmol). The mixture was heated at 65 °C for 16 h then was cooled to room temperature. The product was washed with Et_2O and the solvent was removed under reduced pressure to give amide **14a** (368 mg, 26%) as a pale yellow powder; m.p. 238–240 °C; FT-IR ν_{max} (film)/cm^{-1}: 3017, 1643 (C=O), 1526, 1401, 1383, 1362, 1257, 1239, 1156, 1041, 900, 852, 798; ^{1}H NMR (400 MHz, D_2O) δ = 9.13–9.05 (2H, m, 2 x CH), 8.34 (1H, d, *J* = 6.0 Hz, CH), 8.08–8.03 (3H, m, 3 x CH), 6.07 (2H, s, CH_2), 3.23 (3H, s, CH_3), 2.95 (3H, s, CH_3); ^{13}C NMR (100 MHz, D_2O) δ = 165.2 (C=O), 150.4 (CH), 148.3 (CH), 137.5 (C), 136.6 (CH), 135.9 (C), 130.6 (C), 129.2 (CH), 122.8 (CH), 120.0 (CH), 58.8 (CH_2), 36.4 (CH_3), 35.9 (CH_3); HRMS *m/z* (ES) Found: M^+, 249.0789. $C_{13}H_{14}{}^{35}ClN_2O$ requires M^+ 249.0789; M^+, 251.0764. $C_{13}H_{14}{}^{37}ClN_2O$ requires M^+ 251.0760; LRMS *m/z* (ES) 249.1 (100%, M^+ for ^{35}Cl), 251.1 (30%, M^+ for ^{37}Cl).

6-Bromo-1-[(dimethylcarbamoyl)methyl]quinolin-1-ium bromide (14b)

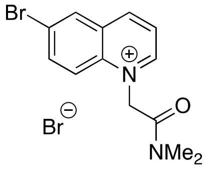

14b

To a stirred solution of 6-bromoquinoline (2 mL, 14.4 mmol) in PhMe (18 mL) was added 2-bromo-*N*,*N*-dimethylacetamide[5] (1.7 mL, 15.9 mmol). The mixture was heated at 65 °C for 16 h then was cooled to room temperature. The mixture was filtered, washed with Et_2O (100 mL) and was dried in air for 30 min to give amide **14b** (2.4 g, 45%) as a pale orange amorphous solid; m.p. 248–250 °C; FT-IR ν_{max} (film)/cm^{-1} 3072, 3013, 1642 (C=O), 1592, 1553, 1403, 1383, 1358, 1263, 1155, 887, 845, 798; ^{1}H NMR (400 MHz, D_2O) δ = 9.21 (1H, d, *J* = 6.0

Hz, CH), 9.16 (1H, d, J = 8.5 Hz, CH), 8.62 (1H, d, J = 2.0 Hz, CH), 8.30 (1H, dd, J = 9.5, 2.0 Hz, CH), 8.15 (1H, dd, J = 8.5, 6.0 Hz, CH), 8.07 (1H, d, J = 9.5 Hz, CH), 6.15 (2H, s, CH_2), 3.31 (3H, s, CH_3), 3.03 (3H, s, CH_3); ^{13}C NMR (100 MHz, D_2O) δ = 165.2 (C=O), 150.5 (CH), 148.2 (CH), 139.2 (CH), 137.8 (C), 132.6 (CH), 130.9 (C), 124.0 (C), 122.7 (CH), 119.8 (CH), 58.7 (CH_2), 36.4 (CH_3), 35.9 (CH_3); HRMS *m/z* (ES) Found: M^+, 293.0281. $C_{13}H_{14}{}^{79}BrN_2O$ requires M^+ 293.0284; Found M^+, 295.0260. $C_{13}H_{14}{}^{81}BrN_2O$ requires M^+ 295.0264; LRMS *m/z* (ES) 293.0 (100%, M^+ for ^{79}Br), 294.0 (15%), 295.0 (100%, M^+ for ^{81}Br), 296.0 (15%).

5-Chloro-*N*,*N*,13-trimethyl-12,14-dioxo-1,13-diazatetracyclo[8.6.0.0^{2,7}.0^{11,15}]hexadeca-2(7),3,5,8-tetraene-16-carboxamide (15a)

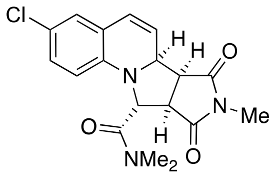

15a

To a stirred suspension of compound **14a** (200 mg, 0.6 mmol) in MeOH (4 mL) was added *N*-methylmaleimide (67 mg, 0.6 mmol) followed by Et_3N (0.08 mL, 0.6 mmol). The mixture was heated under reflux for 1 h and was then cooled to room temperature. The solvent was removed under reduced pressure and the crude product was purified by column chromatography on silica gel, eluting with pentane-EtOAc (1:1), to give amide **15a** (143 mg, 66%) as a yellow powder; m.p. 182–184 °C; R_f 0.17 [petrol-EtOAc (1:1)]; FT-IR ν_{max} /cm^{-1}: 2943, 2814, 1696 (C=O), 1653 (C=O), 1489, 1383, 1292, 1126, 1059, 870, 812, 759; ^{1}H NMR (400 MHz, DMSO) δ = 7.00–6.94 (2H, m, CH), 6.35 (1H, dd, J = 10.0, 2.5 Hz, CH), 6.21 (1H, d, J = 8.5 Hz, CH), 6.06 (1H, dd, J = 10.0, 2.5 Hz, CH), 5.11 (1H, dt, J = 7.0, 2.5 Hz, CH), 4.87 (1H, s, CH), 3.65-3.52 (2H, m, CH), 3.28 (3H, s, CH_3), 2.88 (3H, s, CH_3), 2.84 (3H, s, CH_3); ^{13}C NMR (100 MHz, DMSO) δ = 177.3 (C=O), 175.8 (C=O), 169.1 (C=O), 140.4 (C), 128.4 (CH), 126.9 (CH), 125.5 (CH), 123.9 (C), 121.5 (C), 123.0 (CH), 111.3 (CH), 60.6 (CH), 59.2 (CH), 47.5 (CH), 47.5 (CH), 37.1 (CH_3), 35.9 (CH_3), 25.5 (CH_3); HRMS *m/z* (ES) Found: MH^+, 360.1110. $C_{18}H_{19}{}^{35}ClN_3O_3$ requires MH^+ 360.1109; MH^+,

362.1091. $C_{18}H_{19}{}^{37}ClN_3O_3$ requires MH^+ 362.1080; LRMS *m/z* (ES) 360.1 (100%, MH^+ for ^{35}Cl), 362.1 (30%, MH^+ for ^{37}Cl). CCDC 1907020.

5-Bromo-*N*,*N*,13-trimethyl-12,14-dioxo-1,13-diazatetracyclo[8.6.0.0^{2,7}.0^{11,15}]hexadeca-2(7),3,5,8-tetraene-16-carboxamide (15b)

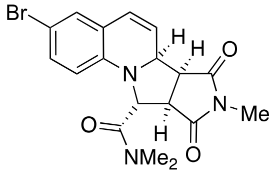

15b

To a stirred suspension of amide **14b** (200 mg, 0.54 mmol) in MeOH (3 mL) was added *N*-methylmaleimide (60 mg, 0.54 mmol) followed by Et_3N (0.08 mL, 0.54 mmol). The mixture was heated under reflux for 1 h and was then cooled to room temperature. The solvent was removed under reduced pressure and the crude product was purified by column chromatography on silica gel, eluting with EtOAc, to give amide **15b** (145 mg, 67%) as an off-white amorphous solid; m.p. 196–198 °C; R_f 0.19 [EtOAc]; FT-IR ν_{max} (film)/cm^{-1} 3011, 2935, 1695 (C=O), 1645 (C=O), 1587, 1486, 1439, 1383, 1288, 1149, 817, 745; ^{1}H NMR (400 MHz, $CDCl_3$) δ = 7.08 (1H, dd, *J* = 8.5, 2.0 Hz, CH), 7.02 (1H, d, *J* = 2.0 Hz, CH), 6.29 (1H, dd, *J* = 10.0, 2.0 Hz, CH), 6.13 (1H, d, *J* = 10.0 Hz, CH), 5.96 (1H, d, *J* = 8.5 Hz, CH), 5.36 (1H, d, *J* = 8.0 Hz, CH), 4.85 (1H, s, CH), 3.48 (1H, t, *J* = 8.0 Hz, CH), 3.41–3.33 (4H, m, CH & CH_3), 3.03 (3H, s, CH_3), 2.97 (3H, s, CH_3); ^{13}C NMR (100 MHz, $CDCl_3$) δ = 177.2 (C=O), 175.7 (C=O), 169.0 (C=O), 140.8 (C), 131.3 (CH), 129.7 (CH), 125.4 (CH), 124.3 (C), 122.9 (CH), 111.7 (CH), 110.6 (C), 60.6 (CH), 59.2 (CH), 47.6 (CH), 47.5 (CH), 37.0 (CH_3), 35.9 (CH_3), 25.5 (CH_3); HRMS *m/z* (ES) Found: MH^+, 404.0593. $C_{18}H_{19}{}^{79}BrN_3O_3$ requires MH^+ 404.0604; Found MH^+, 406.0584. $C_{18}H_{19}{}^{81}BrN_3O_3$ requires M^+ 406.0584; LRMS *m/z* (ES) 404.1 (100%, MH^+ for ^{79}Br), 405.1 (20%), 406.1 (100%, MH^+ for ^{81}Br), 407.1 (20%), 426.0 (30%, MNa^+ for ^{79}Br), 428.0 (30%, MNa^+ for ^{81}Br).

***N*,*N*,13-Trimethyl-12,14-dioxo-5-phenyl-1,13-diazatetracyclo[8.6.0.0^{2,7}.0^{11,15}]hexadeca-2(7),3,5,8-tetraene-16-carboxamide (16)**

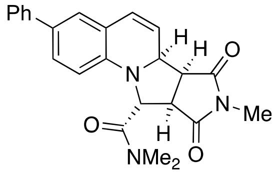

16

The amide **15b** (91 mg, 0.225 mmol), phenylboronic acid (36 mg, 0.296 mmol), tetrakis(triphenylphosphine)palladium(0) (14 mg, 0.012 mmol) and Na_2CO_3 (105 mg, 0.988 mmol) in PhMe (0.2 mL), H_2O (0.2 mL), and EtOH (0.1 mL) were heated at 75 °C for 16 h. After cooling to room temperature, the mixture was filtered through a pad of celite. The filtrate was diluted with water (10 mL) and CH_2Cl_2 (25 mL). The organic layer was separated and was washed with brine (10 mL), dried over anhydrous $MgSO_4$ and filtered. The solvent was removed under reduced pressure. The crude product was purified by column chromatography on silica gel, eluting with petrol–EtOAc (1:4), to give amide **16** (55 mg, 61%) as an off-white amorphous solid; m.p. 200–202 °C; R_f 0.17 [petrol-EtOAc (1:3)]; FT-IR ν_{max} (film)/cm^{-1} 3061, 2960, 2822, 1708 (C=O), 1642 (C=O), 1482, 1431, 1378, 1288, 1131, 769, 703; ^{1}H NMR (400 MHz, $CDCl_3$) δ = 7.47 (2H, dd, *J* = 7.5, 1.0 Hz, 2 × CH), 7.37 (2H, t, *J* = 7.5 Hz, 2 × CH), 7.30–7.23 (2H, m, 2 × CH), 7.17 (1H, d, *J* = 2.0 Hz, CH), 6.45 (1H, dd, *J* = 10.0, 2.0 Hz, CH), 6.21 (1H, d, *J* = 8.0 Hz, CH), 6.14 (1H, dd, *J* = 10.0, 2.0 Hz, CH), 5.42 (1H, dt, *J* = 8.0, 2.0 Hz, CH), 4.98 (1H, s, CH), 3.51 (1H, t, *J* = 8.0 Hz, CH), 3.43 – 3.37 (4H, m, CH & CH_3), 3.05 (3H, s, CH_3), 2.96 (3H, s, CH_3); ^{13}C NMR (100 MHz, $CDCl_3$, one CH missing/overlapping) δ = 177.4 (C=O), 175.9 (C=O), 169.4 (C=O), 141.2 (C), 141.0 (C), 131.9 (C), 128.7 (CH), 127.7 (CH), 126.42 (CH), 126.37 (CH), 126.1 (CH), 122.7 (C), 121.9 (CH), 110.5 (CH), 60.8 (CH), 59.4 (CH), 47.9 (CH), 47.6 (CH), 37.1 (CH_3), 35.9 (CH_3), 25.4 (CH_3); HRMS *m/z* (ES) Found: MH$^+$, 402.1821. $C_{24}H_{24}N_3O_3$ requires MH$^+$ 402.1812; LRMS *m/z* (ES) 261.6 (15%), 402.2 (100%, MH$^+$), 424.2 (30%, MNa$^+$).

3. Single crystal X-ray data

3.1 Data for ester **7c**

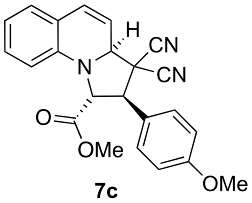

7c

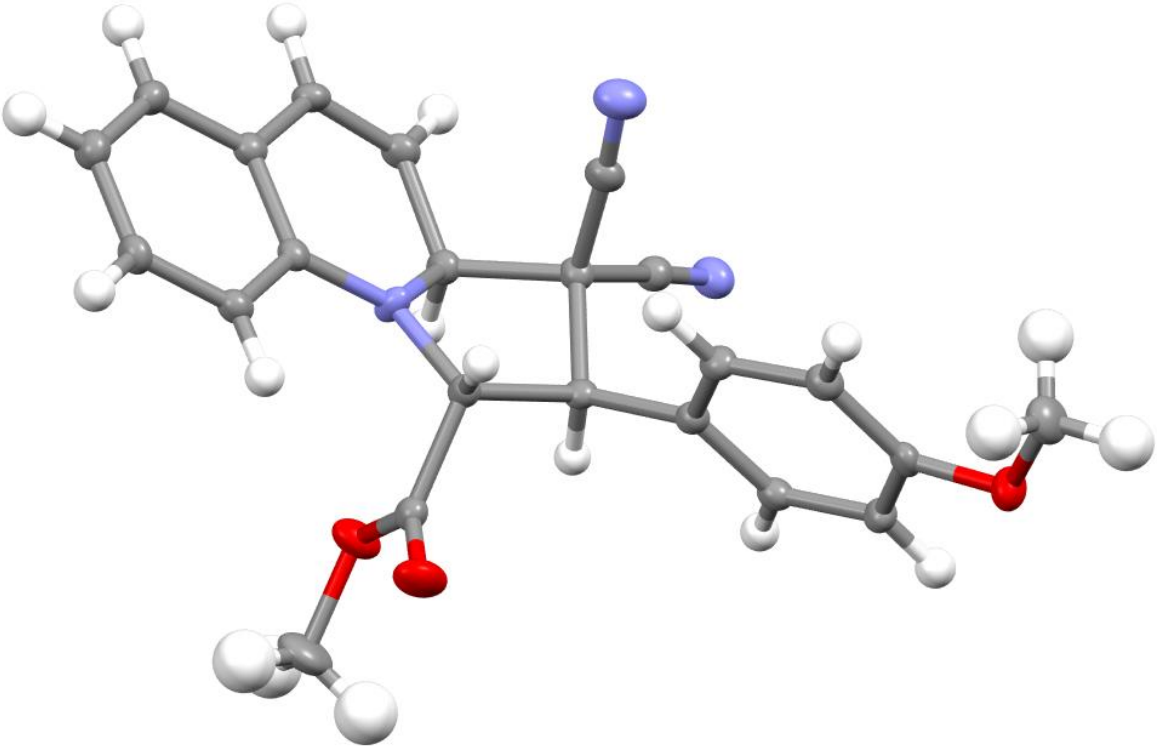

CCDC 1907018

Table S1 Crystal data and structure refinement for 7c (oic289k_0m_a).

Identification code	oic289k_0m_a
Empirical formula	$C_{23}H_{19}N_3O_3$
Formula weight	385.41
Temperature/K	100
Crystal system	monoclinic
Space group	Cc
a/Å	12.810(3)
b/Å	12.661(3)
c/Å	12.025(3)
α/°	90
β/°	95.874(8)
γ/°	90
Volume/Å^3	1940.1(8)
Z	4
ρ_{calc}g/cm^3	1.319
μ/mm^{-1}	0.089
F(000)	808.0
Crystal size/mm^3	0.379 × 0.267 × 0.211
Radiation	MoKα (λ = 0.71073)
2Θ range for data collection/°	4.536 to 55.28
Index ranges	-16 ≤ h ≤ 16, -16 ≤ k ≤ 16, -15 ≤ l ≤ 15
Reflections collected	31377
Independent reflections	4501 [R_{int} = 0.0533, R_{sigma} = 0.0369]
Data/restraints/parameters	4501/2/264
Goodness-of-fit on F^2	1.166
Final R indexes [I>=2σ (I)]	R_1 = 0.0382, wR_2 = 0.1021
Final R indexes [all data]	R_1 = 0.0493, wR_2 = 0.1210
Largest diff. peak/hole / e Å^{-3}	0.26/-0.35

Table S2 Fractional Atomic Coordinates (×10[4]) and Equivalent Isotropic Displacement Parameters (Å^2×10^3) for oic289k_0m_a. U_{eq} is defined as 1/3 of of the trace of the orthogonalised U_{IJ} tensor.

Atom	*x*	*y*	*z*	U(eq)
O1	3935.0(19)	7936.5(17)	5468(2)	30.5(6)
O2	2836.1(19)	7027.2(17)	6445(2)	26.4(5)
O3	8956.5(16)	5996.3(19)	5955.5(18)	24.2(5)
N1	2904(2)	5349.0(19)	5014(2)	18.6(5)
N2	4693(2)	3492(2)	4026(2)	28.9(6)
N3	4823(2)	3142(2)	7654(2)	25.7(6)
C1	2137(2)	5463(2)	4124(2)	15.5(6)
C2	1988(2)	6418(2)	3546(2)	18.4(6)
C3	1194(2)	6500(2)	2674(2)	19.8(6)
C4	559(3)	5643(2)	2342(3)	22.1(6)
C5	716(2)	4694(2)	2910(2)	19.7(6)
C6	1487(2)	4594(2)	3804(2)	16.5(6)
C7	1646(2)	3611(2)	4428(2)	18.3(6)
C8	2329(2)	3539(2)	5336(2)	19.0(6)
C9	2970(2)	4456(2)	5769(2)	16.9(6)
C10	4185(2)	4282(2)	5907(2)	16.4(6)
C11	4578(2)	5451(2)	6068(2)	15.5(5)
C12	3796(2)	6056(2)	5219(2)	16.1(6)
C13	4499(2)	3815(2)	4864(3)	19.7(6)
C14	4551(2)	3623(2)	6876(2)	18.7(6)
C15	5736(2)	5609(2)	5995(2)	16.7(6)
C16	6168(2)	5595(2)	4978(2)	18.5(6)
C17	7246(2)	5692(2)	4934(2)	18.7(6)
C18	7905(2)	5836(2)	5913(2)	17.7(6)
C19	7479(2)	5859(2)	6934(2)	18.8(6)
C20	6411(2)	5741(2)	6969(2)	17.5(6)

C21	3539(2)	7123(2)	5708(2)	18.0(6)
C22	2559(3)	8000(3)	6968(3)	37.2(9)
C23	9447(3)	5808(3)	4957(3)	28.9(7)

Table S3 Anisotropic Displacement Parameters (Å^2×10^3) for oic289k_0m_a. The Anisotropic displacement factor exponent takes the form: $-2\pi^2[h^2a^{*2}U_{11}+2hka^*b^*U_{12}+\ldots]$.

Atom	U_{11}	U_{22}	U_{33}	U_{23}	U_{13}	U_{12}
O1	35.2(14)	18.1(11)	39.8(14)	-4.4(10)	12.2(11)	-7.5(9)
O2	31.0(12)	20.2(11)	30.5(12)	-2.3(9)	14.9(10)	1.9(9)
O3	16.7(11)	31.7(12)	24.2(11)	-1.5(9)	2.5(9)	1.9(9)
N1	19.6(12)	14.2(11)	20.3(12)	3.4(10)	-5(1)	-4.2(9)
N2	36.9(16)	25.7(14)	25.4(14)	-2.8(12)	9.9(12)	-3.2(12)
N3	31.0(15)	23.8(13)	22.0(14)	0.8(11)	0.2(11)	5.4(11)
C1	15.9(13)	16.1(13)	14.9(13)	-0.6(11)	2.8(11)	2.7(10)
C2	19.3(14)	15.2(13)	20.8(14)	-0.2(11)	2.6(12)	0.9(11)
C3	20.3(15)	18.1(14)	20.6(15)	3.4(11)	0.5(12)	4.1(11)
C4	20.9(15)	22.9(15)	21.8(15)	0.6(12)	-1.8(12)	1.5(12)
C5	17.5(15)	19.5(14)	21.8(15)	-3.0(12)	-0.1(12)	-0.9(11)
C6	16.6(14)	16.1(13)	17.1(14)	-0.7(11)	3.1(11)	0.5(11)
C7	21.6(15)	14.2(13)	18.9(14)	-3.3(11)	1.7(12)	-1.8(11)
C8	22.5(15)	14.2(13)	20.4(14)	1.9(11)	2.9(12)	-0.7(11)
C9	18.7(15)	16.0(13)	15.7(14)	2.0(11)	0.0(11)	0.9(11)
C10	18.9(14)	16.7(13)	13.6(13)	0.7(11)	1.6(11)	1.2(11)
C11	18.3(14)	15.3(13)	12.6(13)	-1.2(10)	0.4(11)	0.3(10)
C12	16.9(14)	14.7(13)	16.1(13)	1.1(10)	-0.9(11)	0.1(10)
C13	23.7(15)	14.9(13)	20.6(14)	0.1(12)	2.8(12)	0.5(11)
C14	20.3(14)	18.3(13)	17.5(14)	-0.4(11)	1.6(11)	-0.2(11)
C15	17.8(15)	15.5(13)	16.6(14)	-0.4(11)	0.5(11)	0.4(11)

C16	21.2(15)	19.4(14)	14.1(13)	-0.1(11)	-1.4(12)	0.3(11)
C17	21.7(15)	17.6(13)	17.4(14)	1.6(11)	4.1(12)	1.0(11)
C18	16.4(14)	13.4(12)	23.1(15)	0.5(11)	0.3(12)	3.4(11)
C19	18.1(15)	20.5(14)	17.1(14)	-1.8(11)	-2.0(12)	2.4(11)
C20	20.5(15)	15.7(13)	16.3(14)	-0.8(11)	2.2(11)	1.5(11)
C21	17.1(14)	17.8(14)	18.1(15)	-0.7(11)	-2.0(11)	0.4(11)
C22	44(2)	28.9(18)	41(2)	-10.1(15)	15.8(18)	9.3(15)
C23	21.6(16)	33.3(17)	33.5(17)	-0.5(14)	10.2(14)	1.3(14)

Table S4 Bond Lengths for oic289k_0m_a.

Atom	Atom	Length/Å	Atom	Atom	Length/Å
O1	C21	1.196(4)	C6	C7	1.456(4)
O2	C21	1.332(4)	C7	C8	1.330(4)
O2	C22	1.444(4)	C8	C9	1.486(4)
O3	C18	1.357(4)	C9	C10	1.563(4)
O3	C23	1.431(4)	C10	C11	1.569(4)
N1	C1	1.384(4)	C10	C13	1.479(4)
N1	C9	1.447(4)	C10	C14	1.470(4)
N1	C12	1.453(4)	C11	C12	1.557(4)
N2	C13	1.138(4)	C11	C15	1.508(4)
N3	C14	1.141(4)	C12	C21	1.523(4)
C1	C2	1.398(4)	C15	C16	1.393(4)
C1	C6	1.409(4)	C15	C20	1.394(4)
C2	C3	1.388(4)	C16	C17	1.393(4)
C3	C4	1.390(5)	C17	C18	1.389(4)
C4	C5	1.387(4)	C18	C19	1.394(4)
C5	C6	1.390(4)	C19	C20	1.381(4)

Table S5 Bond Angles for oic289k_0m_a.

Atom	Atom	Atom	Angle/˚	Atom	Atom	Atom	Angle/˚
C21	O2	C22	115.1(2)	C14	C10	C11	111.4(2)
C18	O3	C23	117.5(2)	C14	C10	C13	110.6(2)
C1	N1	C9	123.9(2)	C12	C11	C10	101.9(2)
C1	N1	C12	123.4(2)	C15	C11	C10	115.0(2)
C9	N1	C12	112.4(2)	C15	C11	C12	117.9(2)
N1	C1	C2	121.6(3)	N1	C12	C11	104.5(2)
N1	C1	C6	118.8(2)	N1	C12	C21	114.5(2)
C2	C1	C6	119.6(3)	C21	C12	C11	109.5(2)
C3	C2	C1	119.5(3)	N2	C13	C10	175.7(3)
C2	C3	C4	121.4(3)	N3	C14	C10	177.3(3)
C5	C4	C3	118.9(3)	C16	C15	C11	122.1(2)
C4	C5	C6	121.0(3)	C16	C15	C20	118.2(3)
C1	C6	C7	118.6(2)	C20	C15	C11	119.8(2)
C5	C6	C1	119.6(2)	C15	C16	C17	121.1(3)
C5	C6	C7	121.8(3)	C18	C17	C16	119.9(3)
C8	C7	C6	121.8(3)	O3	C18	C17	124.5(3)
C7	C8	C9	121.8(3)	O3	C18	C19	116.1(2)
N1	C9	C8	113.3(2)	C17	C18	C19	119.3(3)
N1	C9	C10	99.8(2)	C20	C19	C18	120.2(3)
C8	C9	C10	115.8(2)	C19	C20	C15	121.2(3)
C9	C10	C11	100.6(2)	O1	C21	O2	124.9(3)
C13	C10	C9	108.7(2)	O1	C21	C12	123.8(3)
C13	C10	C11	111.7(2)	O2	C21	C12	111.3(2)
C14	C10	C9	113.4(2)				

Table S6 Hydrogen Atom Coordinates (Å×10^4) and Isotropic Displacement Parameters (Å^2×10^3) for oic289k_0m_a.

Atom	*x*	*y*	*z*	U(eq)
H2	2427	7007	3749	22
H3	1083	7155	2296	24
H4	26	5707	1735	27
H5	289	4102	2684	24
H7	1251	3006	4180	22
H8	2408	2882	5717	23
H9	2739	4680	6503	20
H11	4432	5676	6834	19
H12	4123	6167	4508	19
H16	5719	5518	4303	22
H17	7530	5659	4235	22
H19	7925	5956	7607	23
H20	6132	5751	7671	21
H22A	2226	8480	6398	56
H22B	3193	8331	7338	56
H22C	2069	7849	7523	56
H23A	9206	6337	4393	43
H23B	9259	5101	4671	43
H23C	10210	5858	5123	43

3.2 Data for ester **9**

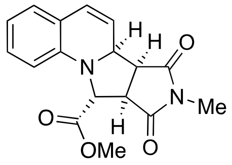

9

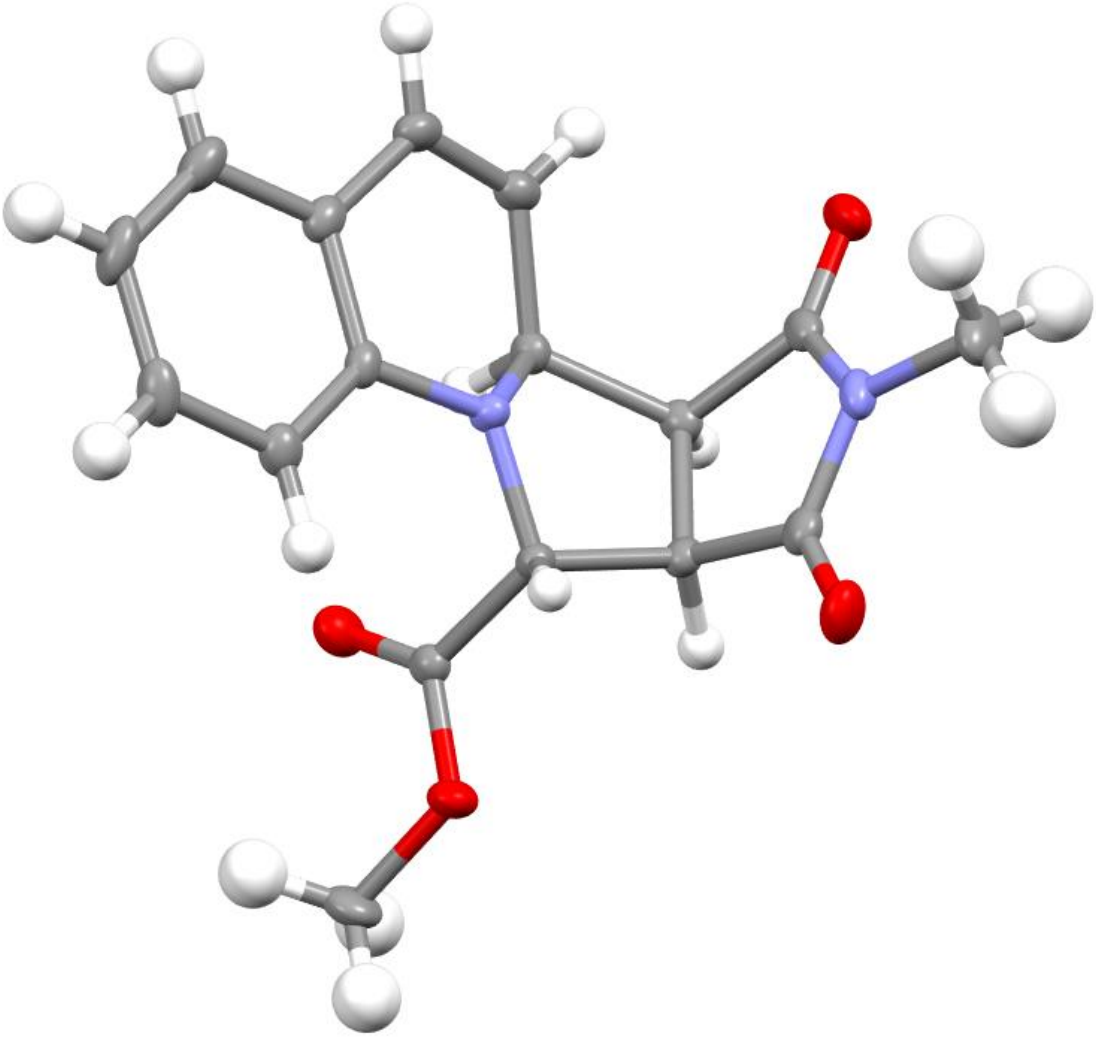

CCDC 1907019

Table S7 Crystal data and structure refinement for 9 (oic292v_0m).

Identification code	oic292v_0m
Empirical formula	$C_{17}H_{16}N_2O_4$
Formula weight	312.32
Temperature/K	99.97
Crystal system	monoclinic
Space group	$P2_1/n$
a/Å	9.8029(10)
b/Å	10.9827(10)
c/Å	14.4853(13)
α/°	90
β/°	108.944(4)
γ/°	90
Volume/Å^3	1475.1(2)
Z	4
ρ_{calc}g/cm^3	1.406
μ/mm^{-1}	0.841
F(000)	656.0
Crystal size/mm^3	0.23 × 0.18 × 0.1
Radiation	CuKα (λ = 1.54178)
2Θ range for data collection/°	10.322 to 133.396
Index ranges	-11 ≤ h ≤ 11, -13 ≤ k ≤ 13, -17 ≤ l ≤ 17
Reflections collected	18953
Independent reflections	2596 [R_{int} = 0.0606, R_{sigma} = 0.0343]
Data/restraints/parameters	2596/0/210
Goodness-of-fit on F^2	1.242
Final R indexes [I>=2σ (I)]	R_1 = 0.0412, wR_2 = 0.1397
Final R indexes [all data]	R_1 = 0.0466, wR_2 = 0.1462
Largest diff. peak/hole / e Å^{-3}	0.31/-0.25

Table S8 Fractional Atomic Coordinates (×10^4) and Equivalent Isotropic Displacement Parameters (Å^2×10^3) for oic292v_0m. U_{eq} is defined as 1/3 of of the trace of the orthogonalised U_{IJ} tensor.

Atom	*x*	*y*	*z*	U(eq)
O1	-1385.9(11)	6064.7(10)	2405.6(9)	30.4(3)
O2	2866.1(12)	7163.1(10)	4647.5(8)	29.0(3)
O3	4207.6(11)	3454.3(10)	2707.0(8)	26.6(3)
O4	5617.8(10)	4813.5(9)	3733.3(8)	23.1(3)
N1	2038.4(12)	3839.9(10)	3565.7(8)	15.6(3)
N2	574.0(14)	6635.3(11)	3698.2(9)	21.6(3)
C1	2209.8(15)	2666.5(13)	3952.3(10)	16.3(3)
C2	3493.8(16)	2255.8(13)	4626.8(10)	20.5(3)
C3	3565.7(18)	1088.4(14)	5004.8(11)	25.8(4)
C4	2381.2(19)	323.0(14)	4738.0(11)	28.4(4)
C5	1090.8(17)	741.7(14)	4080.5(11)	25.0(4)
C6	983.3(16)	1905.4(13)	3676.7(10)	18.8(4)
C7	-359.8(15)	2391.3(13)	2999.7(11)	20.7(3)
C8	-373.8(15)	3402.8(13)	2493.7(10)	19.6(4)
C9	1017.3(14)	4036.4(12)	2576.8(10)	16.4(3)
C10	1038.6(14)	5436.4(13)	2485.6(10)	16.9(3)
C11	-97.2(15)	6062.6(13)	2820.3(11)	19.9(4)
C12	2058.7(16)	6634.0(13)	3955.7(11)	19.9(4)
C13	2479.7(14)	5847.8(12)	3228.8(10)	16.9(3)
C14	3233.9(14)	4675.6(12)	3739.7(10)	15.3(3)
C15	-207(2)	7344.3(15)	4216.4(14)	35.1(4)
C16	4382.5(14)	4227.3(13)	3316.6(10)	17.0(3)
C17	6831.5(16)	4411.7(16)	3438.1(13)	29.2(4)

Table S9 Anisotropic Displacement Parameters (Å^2×10^3) for oic292v_0m. The Anisotropic displacement factor exponent takes the form: -2π^2[h^{2}a*^{2}U$_{11}$+2hka*b*U$_{12}$+…].

Atom	U_{11}	U_{22}	U_{33}	U_{23}	U_{13}	U_{12}
O1	18.6(6)	23.6(6)	48.9(7)	4.7(5)	10.7(5)	3.7(4)
O2	36.8(7)	19.5(6)	28.2(6)	-6.7(4)	7.1(5)	1.9(4)
O3	23.6(6)	28.9(6)	29.2(6)	-8.0(5)	11.4(5)	0.4(4)
O4	15.7(5)	24.4(6)	30.6(6)	-2.0(4)	9.6(4)	-2.8(4)
N1	14.6(6)	14.1(6)	16.8(6)	1.3(5)	3.5(5)	-1.1(4)
N2	26.8(7)	14.8(6)	28.9(7)	2.3(5)	17.0(6)	4.0(5)
C1	22.9(7)	13.3(7)	14.9(7)	-0.8(5)	9.0(6)	1.5(5)
C2	24.8(8)	19.5(8)	16.0(7)	-1.4(6)	5.0(6)	2.2(6)
C3	36.5(9)	22.1(8)	15.7(7)	1.0(6)	4.1(6)	9.9(6)
C4	48.9(10)	13.5(8)	22.9(8)	1.1(6)	11.7(7)	1.9(7)
C5	37.0(9)	16.9(8)	23.2(8)	-2.5(6)	12.5(7)	-6.1(6)
C6	25.1(8)	15.9(7)	17.7(7)	-2.5(5)	10.3(6)	-2.2(6)
C7	18.8(7)	20.9(8)	23.1(8)	-5.8(6)	7.8(6)	-5.1(6)
C8	16.3(7)	21.4(8)	20.6(7)	-3.5(6)	5.3(6)	-1.4(5)
C9	16.4(7)	16.4(7)	15.7(7)	1.0(5)	4.4(5)	1.5(5)
C10	17.1(7)	18.1(8)	15.8(7)	2.9(5)	5.8(5)	1.2(5)
C11	19.4(8)	14.1(7)	28.2(8)	6.6(6)	10.6(6)	2.2(5)
C12	25.6(8)	13.1(7)	22.3(8)	3.0(6)	9.8(6)	0.3(5)
C13	18.0(7)	13.7(7)	20.2(7)	0.3(5)	8.0(6)	-1.3(5)
C14	14.2(7)	14.0(7)	17.6(7)	-1.4(5)	5.0(5)	-1.8(5)
C15	41.5(10)	25.6(9)	50.7(11)	-5.9(8)	32.1(9)	2.6(7)
C16	15.7(7)	16.1(7)	18.2(7)	1.9(6)	4.1(5)	0.8(5)
C17	17.9(8)	36.2(9)	38.2(10)	1.9(7)	15.5(7)	-0.3(7)

Table S10 Bond Lengths for oic292v_0m.

Atom	Atom	Length/Å	Atom	Atom	Length/Å
O1	C11	1.2100(18)	C2	C3	1.387(2)
O2	C12	1.2056(18)	C3	C4	1.383(2)
O3	C16	1.1965(18)	C4	C5	1.391(2)
O4	C16	1.3306(17)	C5	C6	1.395(2)
O4	C17	1.4573(18)	C6	C7	1.463(2)
N1	C1	1.3934(18)	C7	C8	1.328(2)
N1	C9	1.4729(17)	C8	C9	1.5005(19)
N1	C14	1.4448(16)	C9	C10	1.544(2)
N2	C11	1.379(2)	C10	C11	1.5159(19)
N2	C12	1.3802(19)	C10	C13	1.5399(18)
N2	C15	1.4592(19)	C12	C13	1.519(2)
C1	C2	1.394(2)	C13	C14	1.5481(19)
C1	C6	1.411(2)	C14	C16	1.5277(19)

Table S11 Bond Angles for oic292v_0m.

Atom	Atom	Atom	Angle/˚	Atom	Atom	Atom	Angle/˚
C16	O4	C17	115.46(12)	N1	C9	C10	101.85(11)
C1	N1	C9	118.79(11)	C8	C9	C10	119.48(12)
C1	N1	C14	122.16(11)	C11	C10	C9	113.12(11)
C14	N1	C9	109.47(11)	C11	C10	C13	104.22(11)
C11	N2	C12	113.22(12)	C13	C10	C9	105.50(11)
C11	N2	C15	123.01(13)	O1	C11	N2	124.44(14)
C12	N2	C15	123.02(13)	O1	C11	C10	126.79(14)
N1	C1	C2	122.99(13)	N2	C11	C10	108.76(12)
N1	C1	C6	116.97(12)	O2	C12	N2	125.05(14)
C2	C1	C6	119.93(13)	O2	C12	C13	126.69(14)
C3	C2	C1	119.62(14)	N2	C12	C13	108.26(12)

C4	C3	C2	121.40(14)	C10	C13	C14	106.11(11)
C3	C4	C5	118.99(14)	C12	C13	C10	104.91(11)
C4	C5	C6	121.18(14)	C12	C13	C14	109.67(11)
C1	C6	C7	118.17(13)	N1	C14	C13	102.15(10)
C5	C6	C1	118.85(13)	N1	C14	C16	112.77(11)
C5	C6	C7	122.96(13)	C16	C14	C13	112.09(11)
C8	C7	C6	121.36(13)	O3	C16	O4	124.71(13)
C7	C8	C9	119.82(13)	O3	C16	C14	125.24(13)
N1	C9	C8	108.85(11)	O4	C16	C14	110.05(12)

Table S12 Hydrogen Atom Coordinates (Å×10^4) and Isotropic Displacement Parameters (Å^2×10^3) for oic292v_0m.

**Atom**	***x***	***y***	***z***	**U(eq)**
H2	4315	2773	4826	25
H3	4447	808	5457	31
H4	2447	-476	5000	34
H5	268	226	3903	30
H7	-1240	1975	2920	25
H8	-1262	3724	2080	24
H9	1430	3670	2093	20
H10	965	5689	1808	20
H13	3088	6304	2909	20
H14	3678	4824	4456	18
H15A	-917	7866	3752	53
H15B	474	7852	4712	53
H15C	-703	6792	4533	53
H17A	6670	4648	2758	44
H17B	6921	3524	3498	44
H17C	7720	4792	3859	44

3.3 Data for amide **15a**

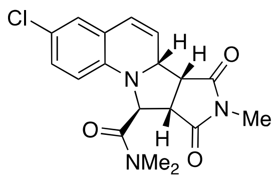

15a

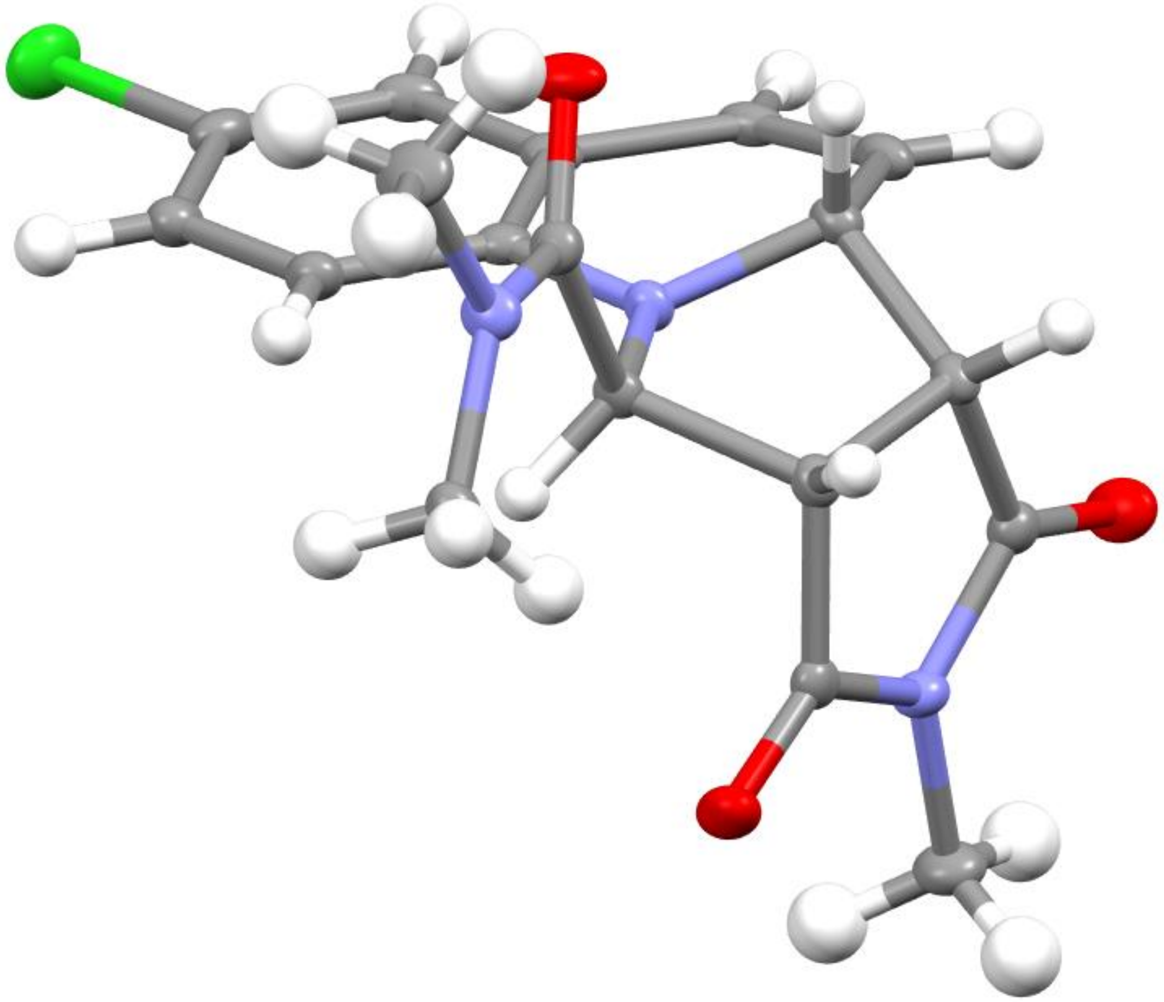

CCDC 1907020

Table S13 Crystal data and structure refinement for 15a (oic300k_0m).

Identification code	oic300k_0m
Empirical formula	$C_{18}H_{18}ClN_3O_3$
Formula weight	359.80
Temperature/K	100
Crystal system	monoclinic
Space group	$P2_1/n$
a/Å	10.2799(17)
b/Å	11.624(2)
c/Å	14.192(3)
α/°	90
β/°	102.689(3)
γ/°	90
Volume/Å^3	1654.5(5)
Z	4
ρ_{calc}g/cm^3	1.445
μ/mm^{-1}	0.254
F(000)	752.0
Crystal size/mm^3	0.5 × 0.4 × 0.4
Radiation	MoKα (λ = 0.71073)
2Θ range for data collection/°	4.462 to 55.054
Index ranges	$-9 \leq h \leq 13$, $-15 \leq k \leq 15$, $-18 \leq l \leq 18$
Reflections collected	22684
Independent reflections	3805 [R_{int} = 0.0452, R_{sigma} = 0.0364]
Data/restraints/parameters	3805/0/229
Goodness-of-fit on F^2	1.034
Final R indexes [I>=2σ (I)]	R_1 = 0.0398, wR_2 = 0.0856
Final R indexes [all data]	R_1 = 0.0593, wR_2 = 0.0950
Largest diff. peak/hole / e Å^{-3}	0.33/-0.38

Table S14 Fractional Atomic Coordinates (×10[4]) and Equivalent Isotropic Displacement Parameters (Å[2]×10[3]) for oic300k_0m. U_{eq} is defined as 1/3 of of the trace of the orthogonalised U_{IJ} tensor.

**Atom**	***x***	***y***	***z***	**U(eq)**
Cl1	2897.9(5)	10883.2(4)	5692.2(3)	27.43(13)
O1	3984.5(12)	7016.9(11)	2306.7(9)	24.5(3)
O2	3373.8(12)	3378.3(10)	4501.6(9)	22.8(3)
O3	-824.9(12)	4617.3(11)	3189.9(10)	25.1(3)
N1	2346.4(13)	6516.7(11)	3508(1)	13.2(3)
N2	1186.6(14)	3940.0(12)	4036(1)	14.8(3)
N3	5507.6(14)	5645.6(12)	2882.9(10)	17.4(3)
C1	1279.9(16)	6331.5(14)	2648.9(12)	14.2(3)
C2	59.1(17)	6994.5(15)	2728.7(12)	16.3(4)
C3	135.3(17)	7898.8(14)	3315.5(12)	16.3(4)
C4	1395.6(16)	8271.9(14)	3929.8(11)	14.6(3)
C5	1525.3(18)	9298.2(14)	4452.2(12)	17.6(4)
C6	2742.3(19)	9596.9(14)	5040.6(12)	18.6(4)
C7	3843.7(18)	8895.9(14)	5113.2(12)	17.6(4)
C8	3740.6(17)	7878.3(14)	4587.2(12)	16.2(4)
C9	2523.2(16)	7555.5(14)	3997.7(11)	13.6(3)
C10	3425.7(16)	5728.8(13)	3483.8(12)	13.2(3)
C11	2646.9(16)	4611.7(14)	3096.9(11)	13.7(3)
C12	2493.0(17)	3883.6(13)	3950.4(12)	14.4(3)
C13	370.2(17)	4518.2(14)	3278.8(12)	15.6(3)
C14	1213.4(16)	5000.2(14)	2621.1(11)	13.9(3)
C15	4334.6(17)	6183.1(14)	2834.7(12)	15.6(3)
C16	6048.8(17)	4755.0(15)	3591.4(12)	17.5(4)
C17	6422.4(19)	6153.4(17)	2339.6(14)	26.1(4)
C18	686.7(19)	3336.8(16)	4783.5(13)	23.2(4)

Table S15 Anisotropic Displacement Parameters (Å^2×10^3) for oic300k_0m. The Anisotropic displacement factor exponent takes the form: -2π^2[h^{2}a*^{2}U$_{11}$+2hka*b*U$_{12}$+…].

Atom	U_{11}	U_{22}	U_{33}	U_{23}	U_{13}	U_{12}
Cl1	34.2(3)	16.9(2)	33.0(3)	-6.42(19)	11.2(2)	1.75(19)
O1	18.3(7)	26.6(7)	30.7(7)	14.1(6)	10.1(6)	5.0(5)
O2	18.0(7)	19.7(6)	28.3(7)	8.0(5)	0.1(5)	1.5(5)
O3	12.8(6)	28.8(7)	34.5(7)	8.4(6)	6.9(6)	2.1(5)
N1	9.9(7)	14.5(7)	15.0(7)	0.3(5)	2.2(5)	2.8(5)
N2	15.6(7)	14.1(7)	16.1(7)	2.1(5)	6.5(6)	0.7(6)
N3	11.5(7)	21.5(7)	20.7(7)	2.2(6)	6.5(6)	1.5(6)
C1	11.9(8)	17.9(8)	13.2(7)	2.7(6)	3.4(6)	1.3(6)
C2	10.1(8)	22.1(9)	16.5(8)	7.3(7)	2.7(6)	1.8(7)
C3	12.4(8)	20.0(8)	18.3(8)	8.2(7)	7.4(7)	5.9(7)
C4	14.2(8)	17.5(8)	13.9(8)	6.0(6)	7.3(6)	3.4(7)
C5	19.6(9)	17.0(8)	19.2(8)	4.7(7)	10.4(7)	6.9(7)
C6	27.5(10)	12.5(8)	17.6(8)	1.5(7)	9.0(7)	0.6(7)
C7	19.2(9)	16.4(8)	16.9(8)	2.3(6)	3.4(7)	0.1(7)
C8	15.0(9)	15.2(8)	18.2(8)	3.1(6)	3.5(7)	2.6(7)
C9	15.0(8)	13.6(8)	13.7(8)	3.4(6)	6.3(6)	1.8(6)
C10	10.2(8)	14.2(8)	15.3(7)	0.4(6)	2.8(6)	3.1(6)
C11	12.2(8)	15.0(8)	14.4(7)	-1.6(6)	3.8(6)	1.9(6)
C12	15.8(8)	11.3(7)	15.6(8)	-2.2(6)	2.6(7)	0.4(6)
C13	15.6(9)	13.8(8)	17.6(8)	-0.5(6)	4.3(7)	-0.6(7)
C14	12.7(8)	18.0(8)	11.1(7)	-0.1(6)	2.9(6)	0.3(7)
C15	11.8(8)	17.4(8)	17.9(8)	0.5(7)	4.1(7)	0.3(6)
C16	14.5(9)	19.0(8)	18.5(8)	-1.4(7)	2.1(7)	3.3(7)
C17	16.0(9)	32.0(11)	33.9(11)	5.6(8)	13.2(8)	1.2(8)
C18	23.7(10)	24.2(9)	24.0(9)	7.4(7)	10.2(8)	-0.4(8)

Table S16 Bond Lengths for oic300k_0m.

Atom	Atom	Length/Å	Atom	Atom	Length/Å
Cl1	C6	1.7469(17)	C1	C14	1.549(2)
O1	C15	1.230(2)	C2	C3	1.333(2)
O2	C12	1.211(2)	C3	C4	1.460(2)
O3	C13	1.213(2)	C4	C5	1.395(2)
N1	C1	1.465(2)	C4	C9	1.413(2)
N1	C9	1.385(2)	C5	C6	1.387(3)
N1	C10	1.445(2)	C6	C7	1.380(2)
N2	C12	1.376(2)	C7	C8	1.390(2)
N2	C13	1.384(2)	C8	C9	1.395(2)
N2	C18	1.456(2)	C10	C11	1.561(2)
N3	C15	1.346(2)	C10	C15	1.542(2)
N3	C16	1.465(2)	C11	C12	1.514(2)
N3	C17	1.465(2)	C11	C14	1.547(2)
C1	C2	1.498(2)	C13	C14	1.514(2)

Table S17 Bond Angles for oic300k_0m.

Atom	Atom	Atom	Angle/°	Atom	Atom	Atom	Angle/°
C9	N1	C1	122.50(13)	C7	C8	C9	120.21(16)
C9	N1	C10	123.21(13)	N1	C9	C4	117.66(15)
C10	N1	C1	108.97(12)	N1	C9	C8	122.46(15)
C12	N2	C13	113.06(14)	C8	C9	C4	119.78(15)
C12	N2	C18	123.28(14)	N1	C10	C11	101.45(12)
C13	N2	C18	123.28(15)	N1	C10	C15	111.49(13)
C15	N3	C16	124.31(15)	C15	C10	C11	113.95(13)
C15	N3	C17	117.23(15)	C12	C11	C10	108.62(13)
C17	N3	C16	117.07(14)	C12	C11	C14	104.79(13)
N1	C1	C2	110.34(13)	C14	C11	C10	106.05(13)
N1	C1	C14	100.88(12)	O2	C12	N2	124.88(16)
C2	C1	C14	118.87(14)	O2	C12	C11	126.40(16)
C3	C2	C1	121.19(15)	N2	C12	C11	108.63(13)

C2	C3	C4	122.02(16)	O3	C13	N2	123.69(16)
C5	C4	C3	122.69(15)	O3	C13	C14	127.23(15)
C5	C4	C9	119.10(15)	N2	C13	C14	109.08(14)
C9	C4	C3	118.21(15)	C11	C14	C1	104.35(13)
C6	C5	C4	120.14(16)	C13	C14	C1	112.46(14)
C5	C6	Cl1	119.91(14)	C13	C14	C11	104.02(13)
C7	C6	Cl1	119.17(14)	O1	C15	N3	122.40(16)
C7	C6	C5	120.91(16)	O1	C15	C10	119.94(15)
C6	C7	C8	119.84(16)	N3	C15	C10	117.65(14)

Table S18 Hydrogen Atom Coordinates (Å×10^4) and Isotropic Displacement Parameters (Å^2×10^3) for oic300k_0m.

Atom	*x*	*y*	*z*	U(eq)
H1	1591	6609	2069	17
H2	-785	6766	2354	20
H3	-655	8313	3335	20
H5	779	9793	4405	21
H7	4670	9108	5522	21
H8	4502	7401	4629	19
H10	3962	5597	4153	16
H11	3079	4180	2638	16
H14	923	4703	1946	17
H16A	6510	5119	4196	26
H16B	6679	4276	3339	26
H16C	5319	4275	3713	26
H17A	5941	6310	1676	39
H17B	7154	5616	2331	39
H17C	6784	6874	2648	39
H18A	751	2505	4692	35
H18B	-247	3548	4742	35
H18C	1220	3552	5420	35

4. References

1. Dolomanov, O.V., Bourhis, L.J., Gildea, R.J, Howard, J.A.K. & Puschmann, H., *J. Appl. Cryst.*, **2009**, *42*, 339.
2. G. M. Sheldrick, *Acta Cryst.*, **2015**, *A71*, 3.
3. G. M. Sheldrick, *Acta Cryst.*, **2015**, *C71*, 3.
4. Grigoryan, J. V; Sargsyan, G. T.; Gyulnazaryan, A. K.; Paronikyan, R. V; Stepanyan, G. M. *Pharm. Chem. J.* **2013**, *47*, 477.
5. Pospíšil, J.; Potáček, M. *Tetrahedron* **2007**, *63*, 337.
6. Yamashita, K.; Tanaka, T.; Hayashi, M. *Tetrahedron* **2005**, *61*, 7981.

5. **NMR spectra** (room temperature in $CDCl_3$ unless stated)

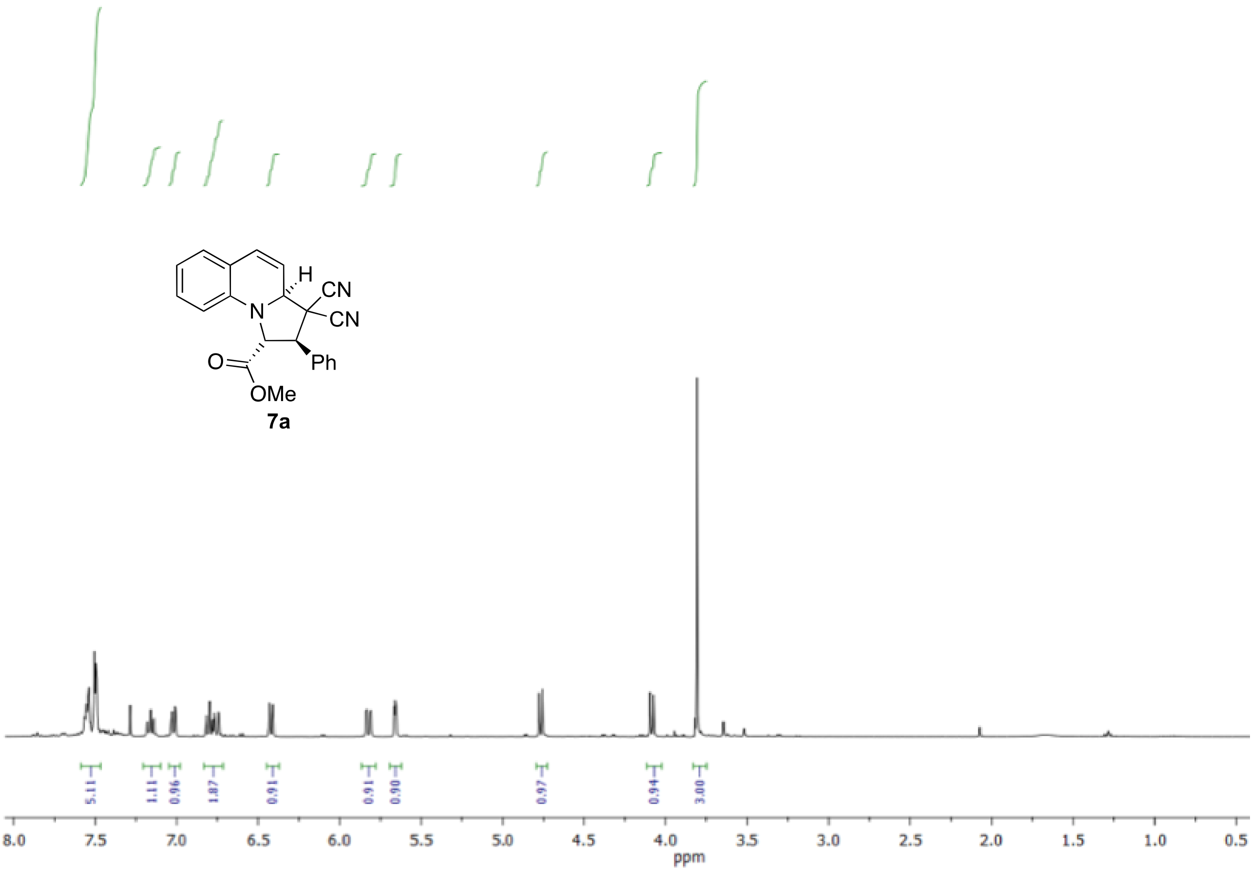

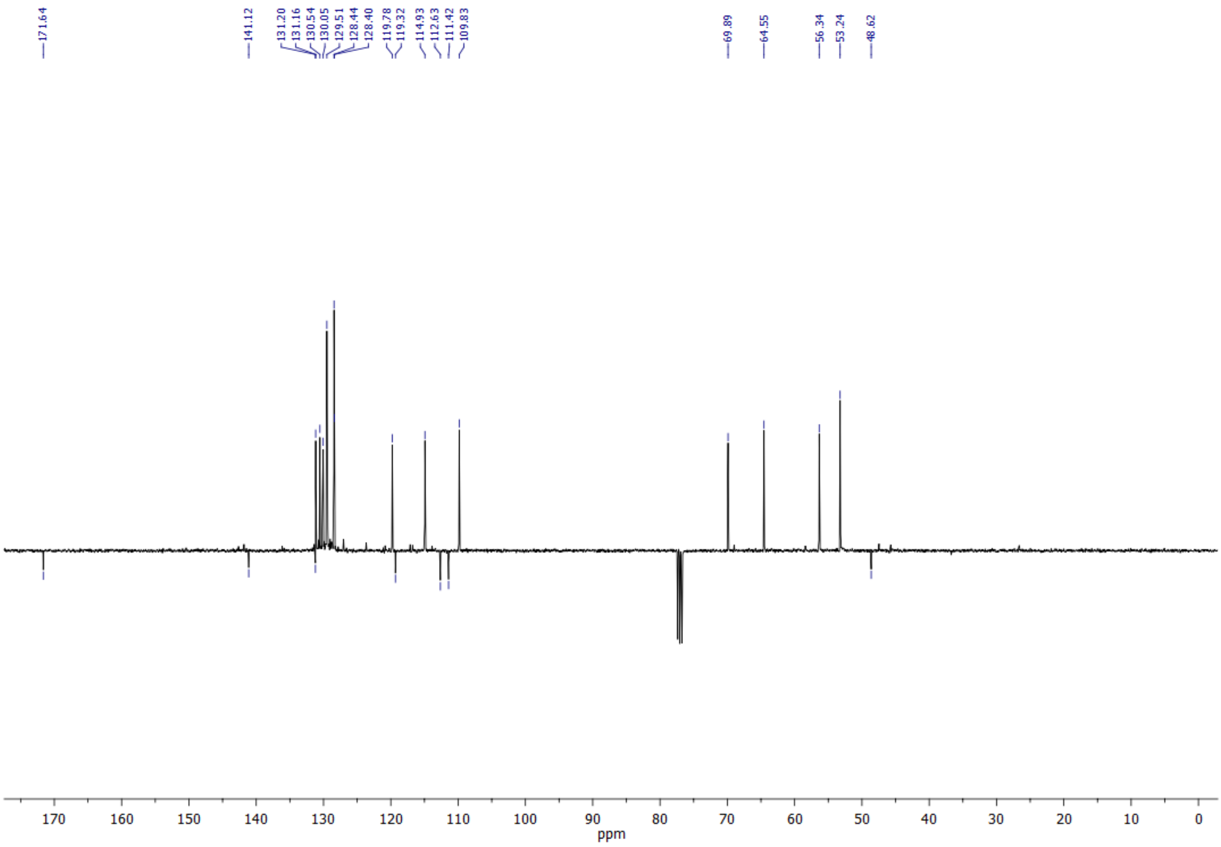

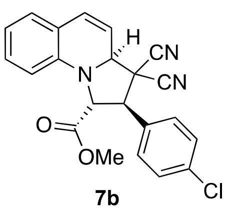
H
CN
CN
N
O
OMe
Cl

7b

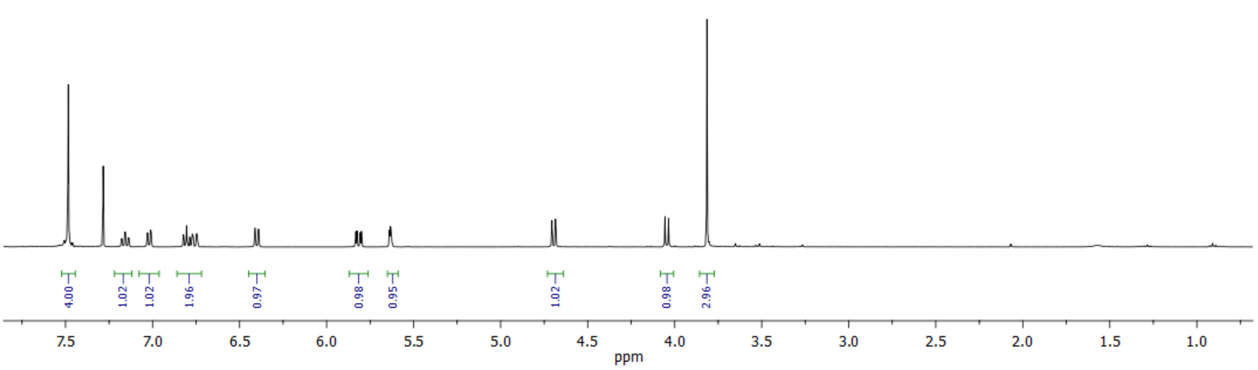
4.00
1.02
1.02
1.96
0.97
0.98
0.95
1.02
0.98
2.96
7.5
7.0
6.5
6.0
5.5
5.0
4.5
4.0
3.5
3.0
2.5
2.0
1.5
1.0
ppm

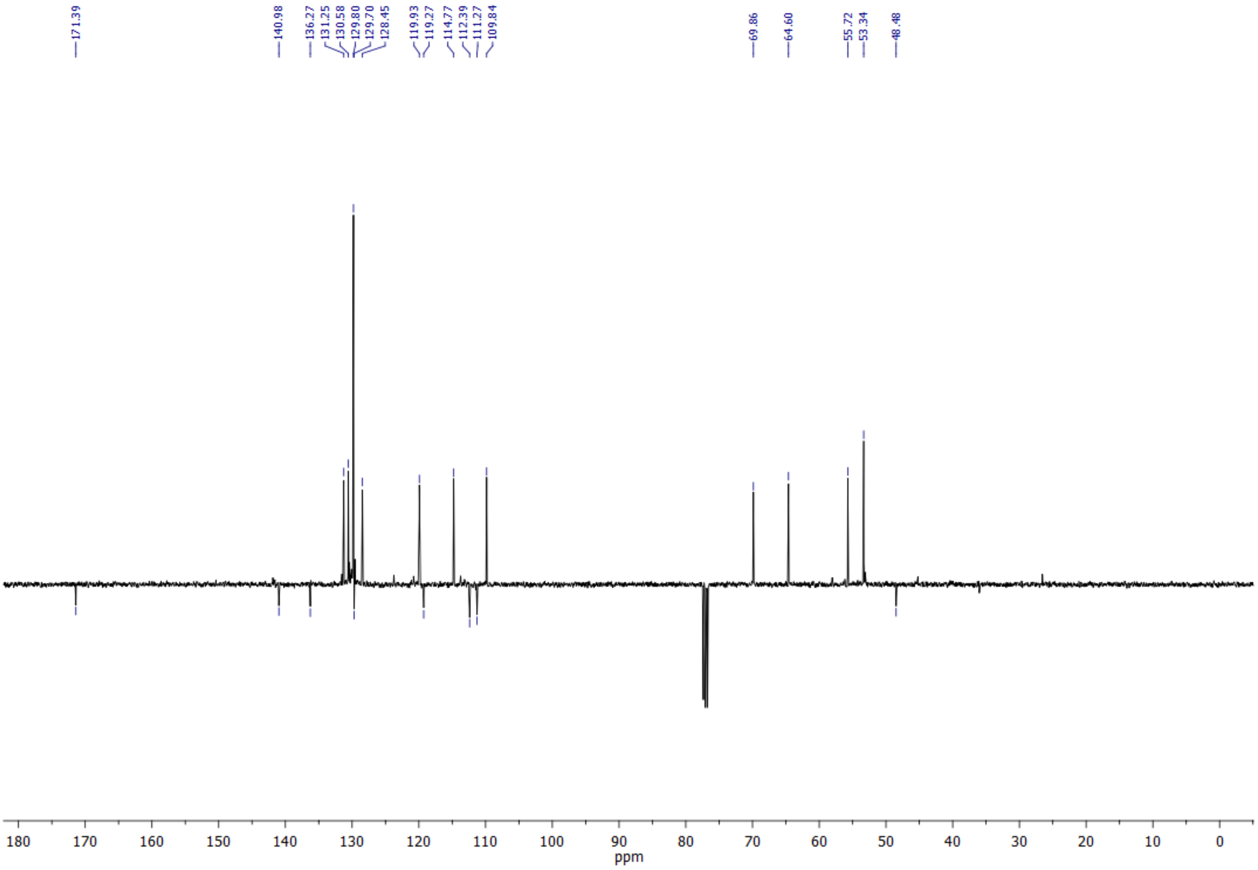
171.39
140.98
136.27
131.25
130.58
129.80
129.70
128.45
119.93
119.27
114.77
112.39
111.27
109.84
69.86
64.60
55.72
53.34
48.48
180
170
160
150
140
130
120
110
100
90
80
70
60
50
40
30
20
10
0
ppm

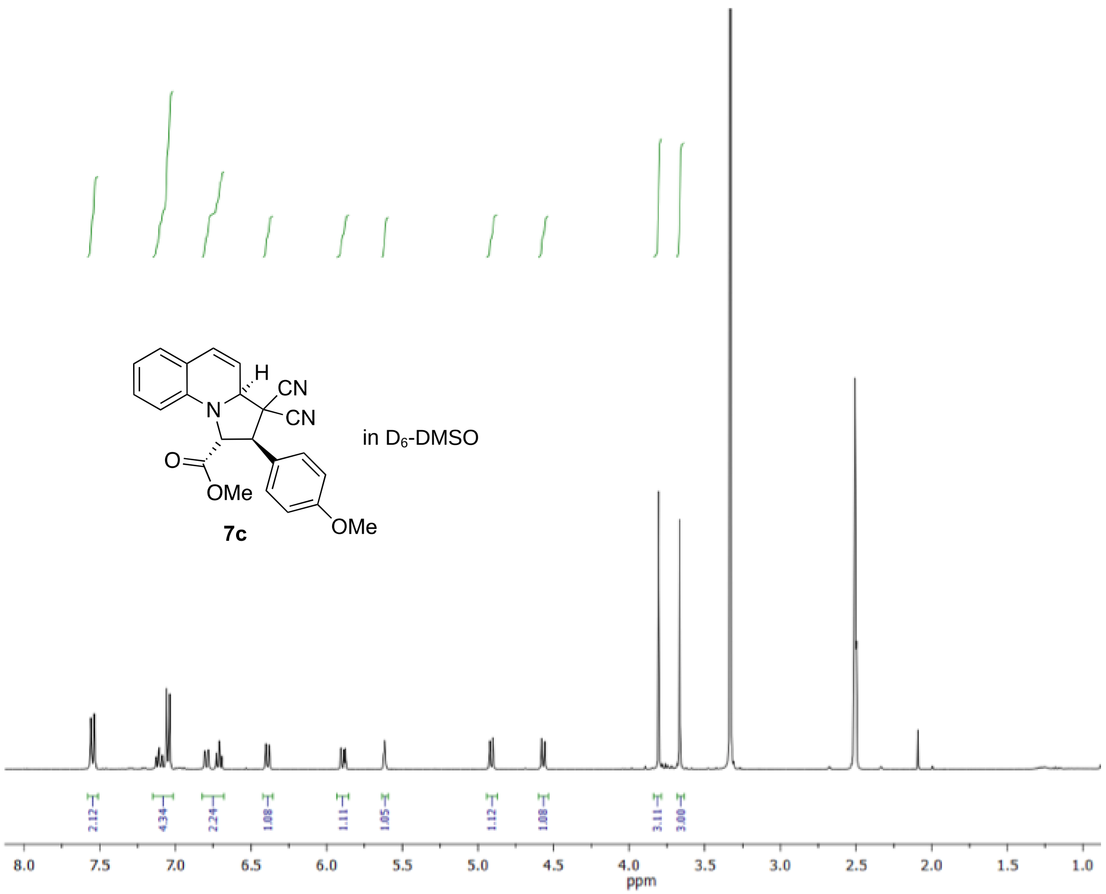
H
CN
CN
N
O
OMe
OMe
7c
in D_6-DMSO
2.12
4.34
2.24
1.08
1.11
1.05
1.12
1.08
3.11
3.00
8.0 7.5 7.0 6.5 6.0 5.5 5.0 4.5 4.0 3.5 3.0 2.5 2.0 1.5 1.0
ppm

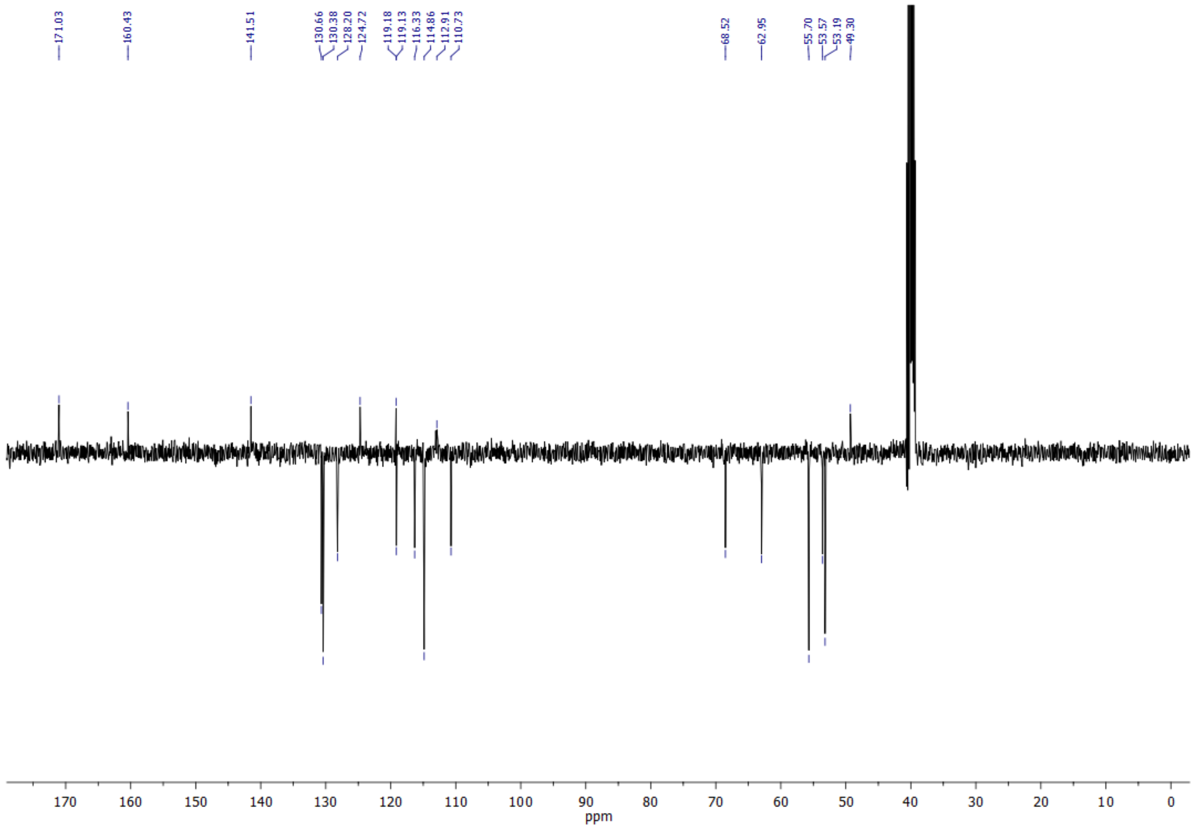
171.03
160.43
141.51
130.66
130.38
128.20
124.72
119.18
119.13
116.33
114.86
112.91
110.73
68.52
62.95
55.70
53.57
53.19
49.30
170 160 150 140 130 120 110 100 90 80 70 60 50 40 30 20 10 0
ppm

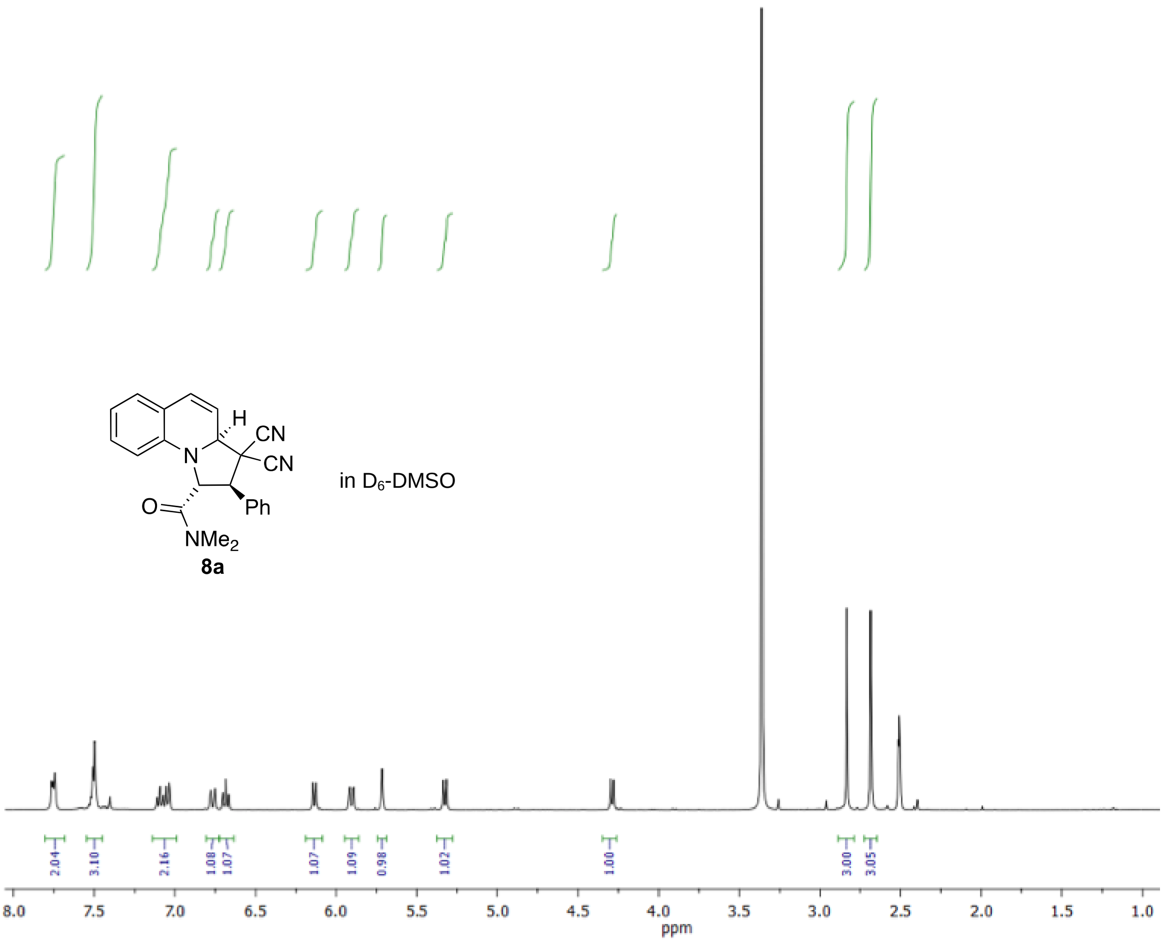
H
CN
CN
N
Ph
O
NMe2
8a
in D6-DMSO
2.04
3.10
2.16
1.08
1.07
1.07
1.09
0.98
1.02
1.00
3.00
3.05
8.0 7.5 7.0 6.5 6.0 5.5 5.0 4.5 4.0 3.5 3.0 2.5 2.0 1.5 1.0
ppm

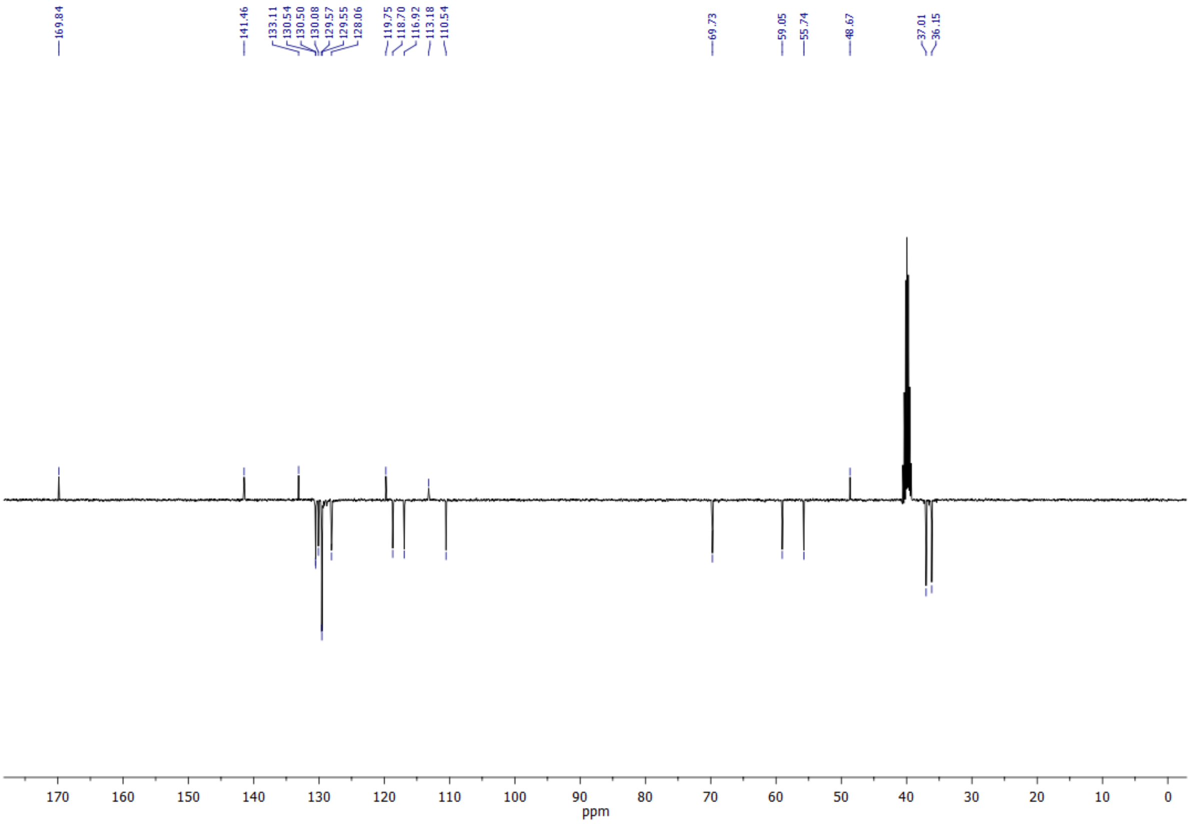
169.84
141.46
133.11
130.54
130.50
130.08
129.57
129.55
128.06
119.75
118.70
116.92
113.18
110.54
69.73
59.05
55.74
48.67
37.01
36.15
170 160 150 140 130 120 110 100 90 80 70 60 50 40 30 20 10 0
ppm

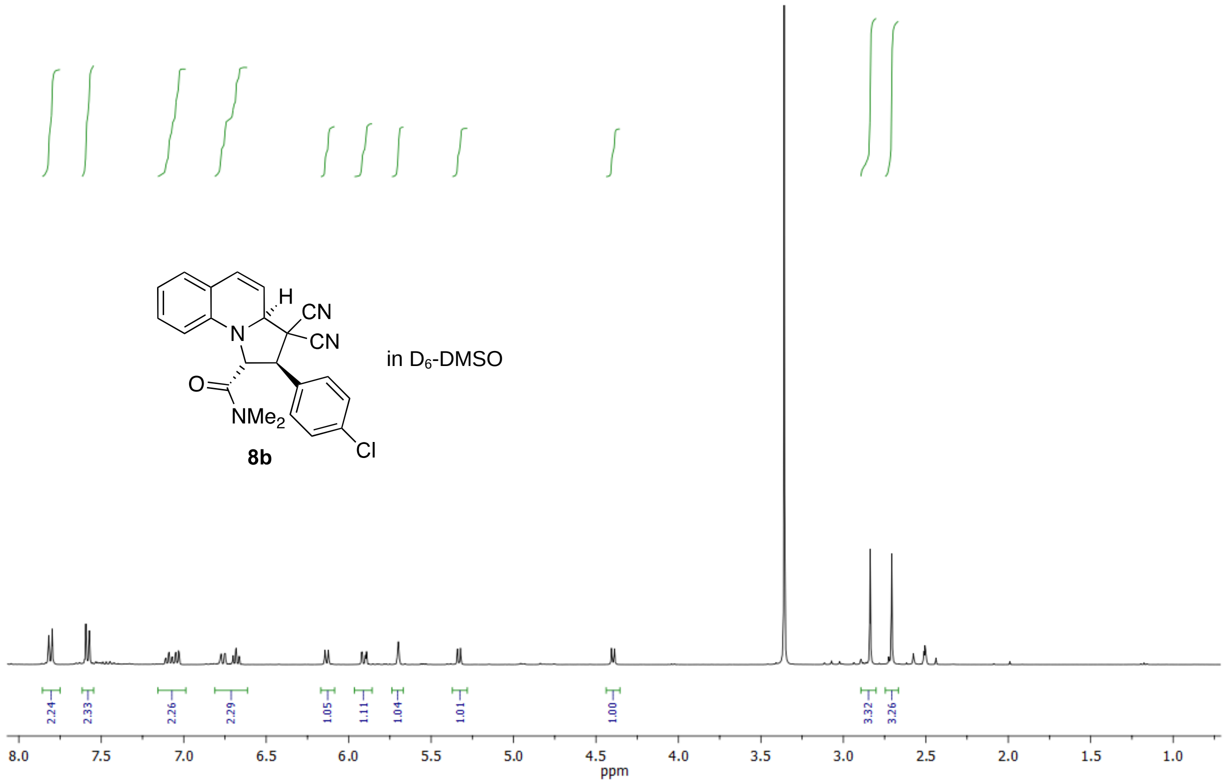

H
CN
CN
N
O
NMe2
Cl
8b
in D6-DMSO
2.24
2.33
2.26
2.29
1.05
1.11
1.04
1.01
1.00
3.32
3.26
ppm

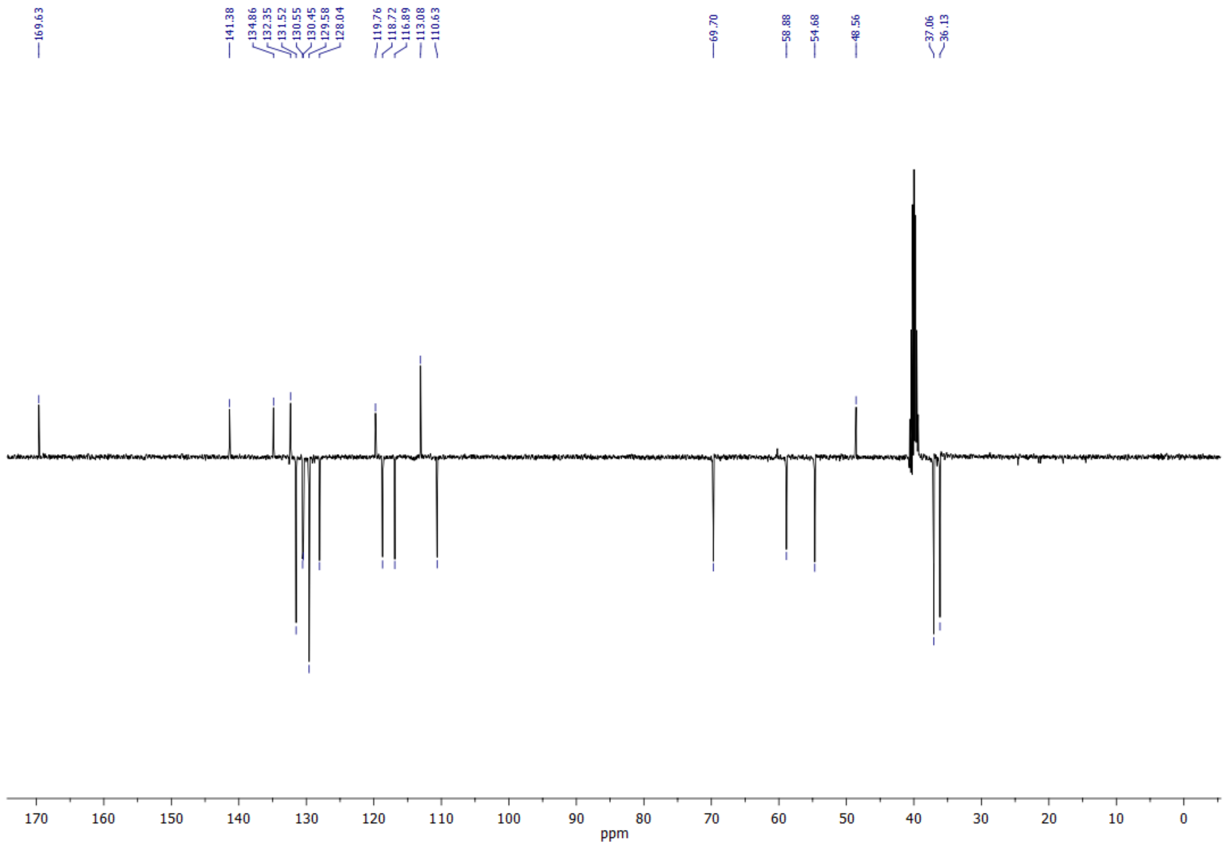

169.63
141.38
134.86
132.35
131.52
130.55
130.45
129.58
128.04
119.76
118.72
116.89
113.08
110.63
69.70
58.88
54.68
48.56
37.06
36.13
ppm

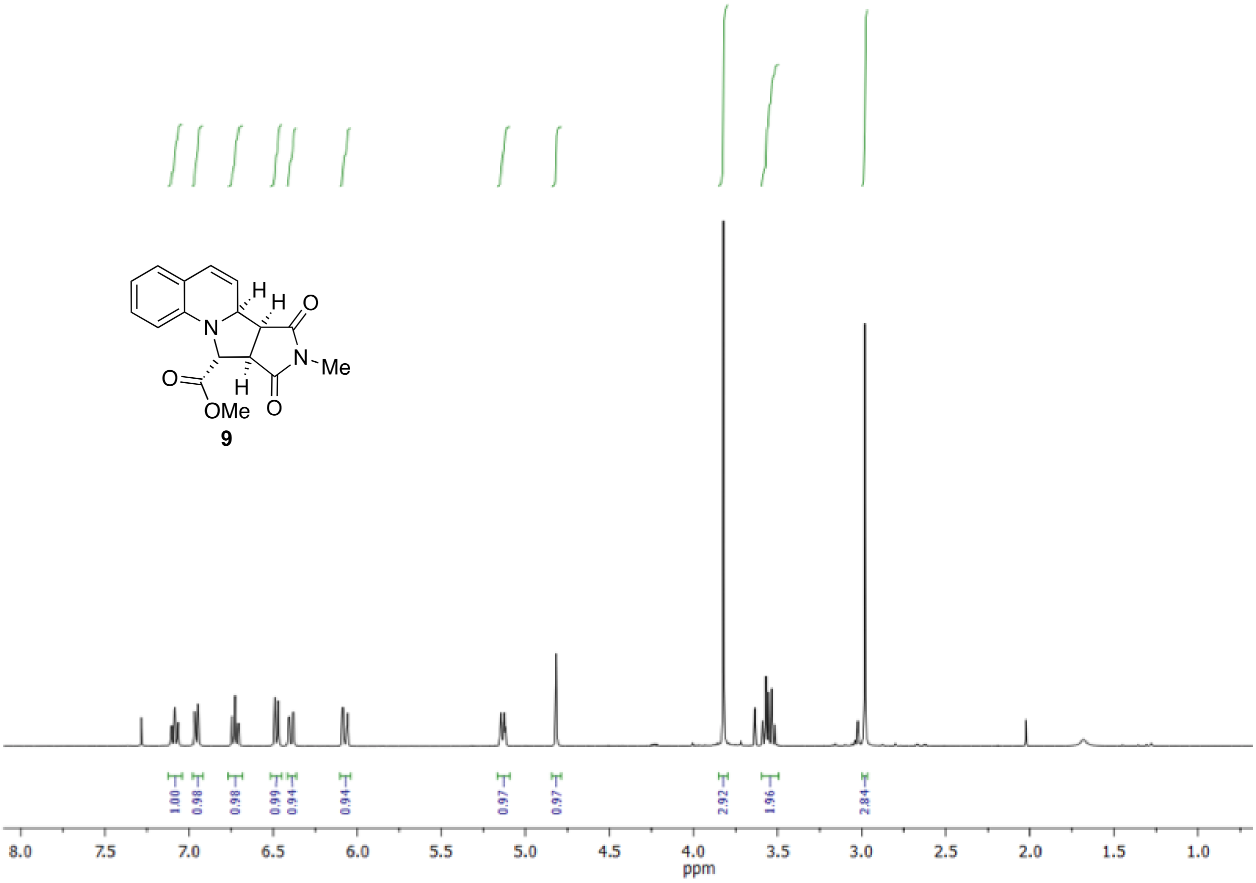
H
H
O
N
N
Me
O
H
O
OMe
9
1.00
0.98
0.98
0.99
0.94
0.94
0.97
0.97
2.92
1.96
2.84
8.0 7.5 7.0 6.5 6.0 5.5 5.0 4.5 4.0 3.5 3.0 2.5 2.0 1.5 1.0
ppm

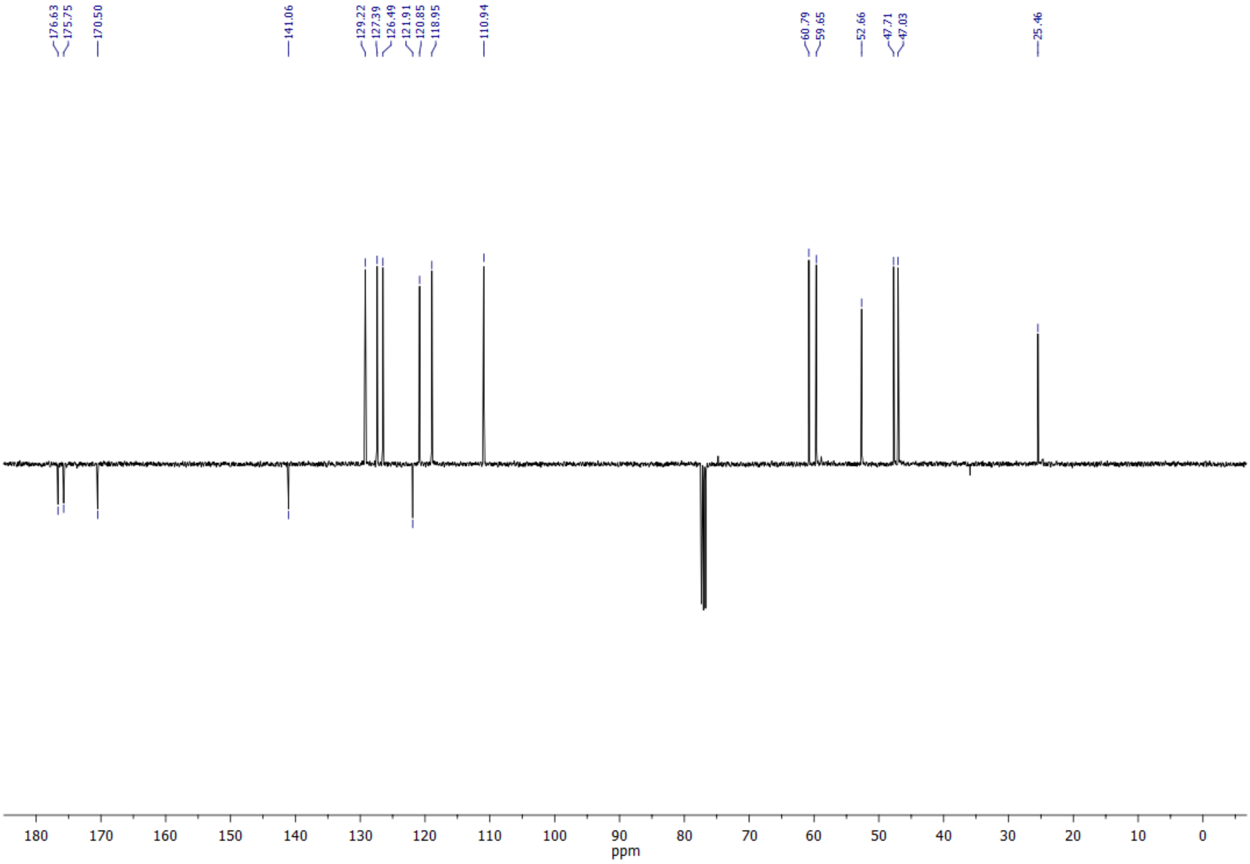
176.63
175.75
170.50
141.06
129.22
127.39
126.49
121.91
120.85
118.95
110.94
60.79
59.65
52.66
47.71
47.03
25.46
180 170 160 150 140 130 120 110 100 90 80 70 60 50 40 30 20 10 0
ppm

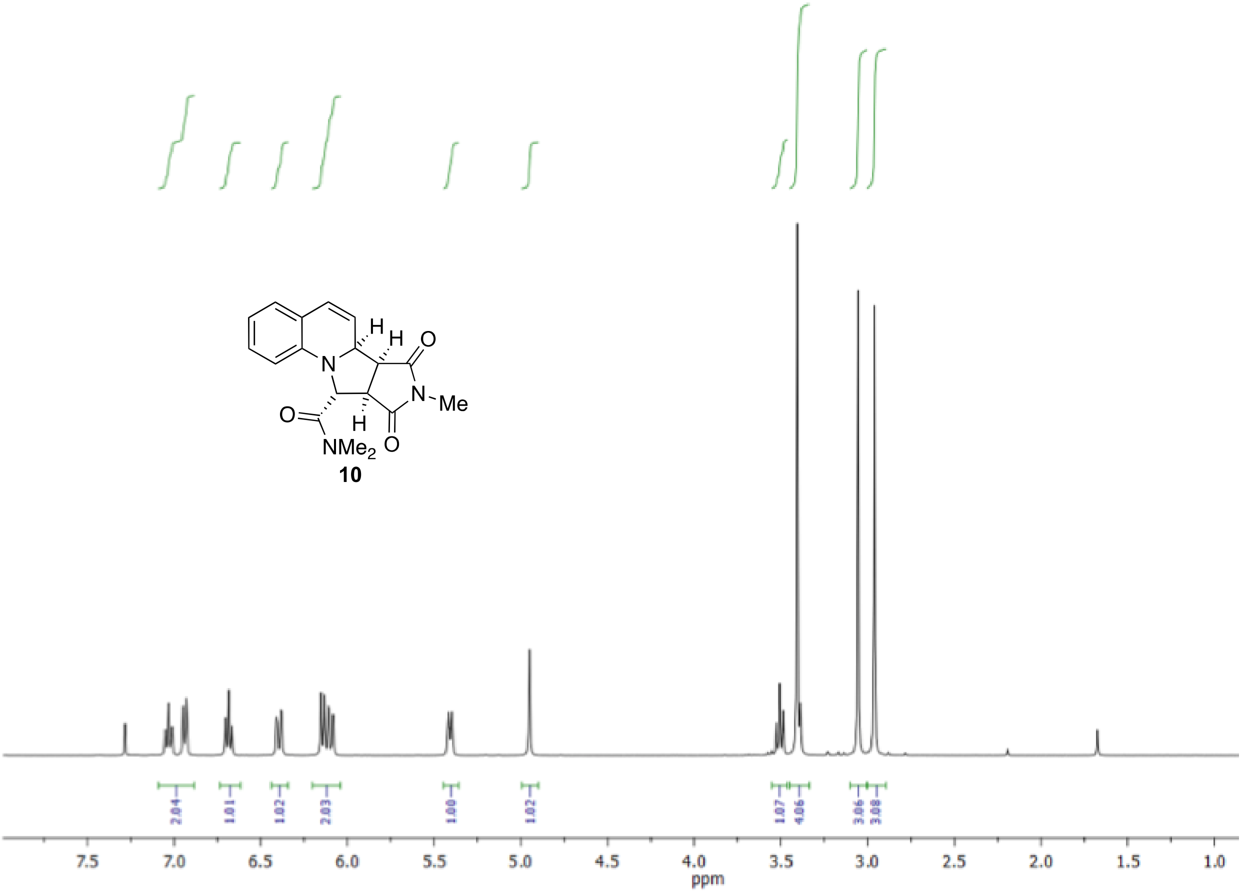
H
H
O
N
N
Me
O
H
NMe$_2$
O
10
2.04
1.01
1.02
2.03
1.00
1.02
1.07
4.06
3.06
3.08
7.5 7.0 6.5 6.0 5.5 5.0 4.5 4.0 3.5 3.0 2.5 2.0 1.5 1.0
ppm

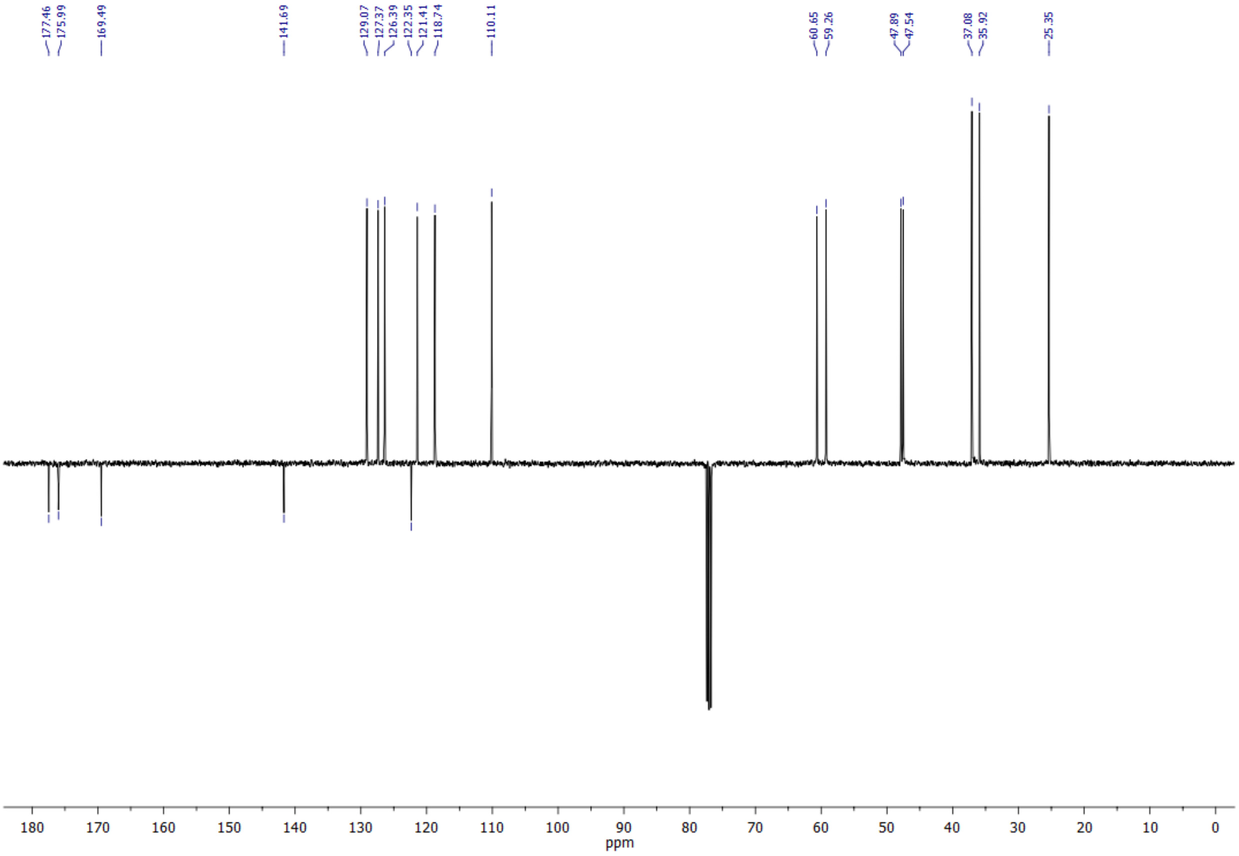
177.46
175.99
169.49
141.69
129.07
127.37
126.39
122.35
121.41
118.74
110.11
60.65
59.26
47.89
47.54
37.08
35.92
25.35
180 170 160 150 140 130 120 110 100 90 80 70 60 50 40 30 20 10 0
ppm

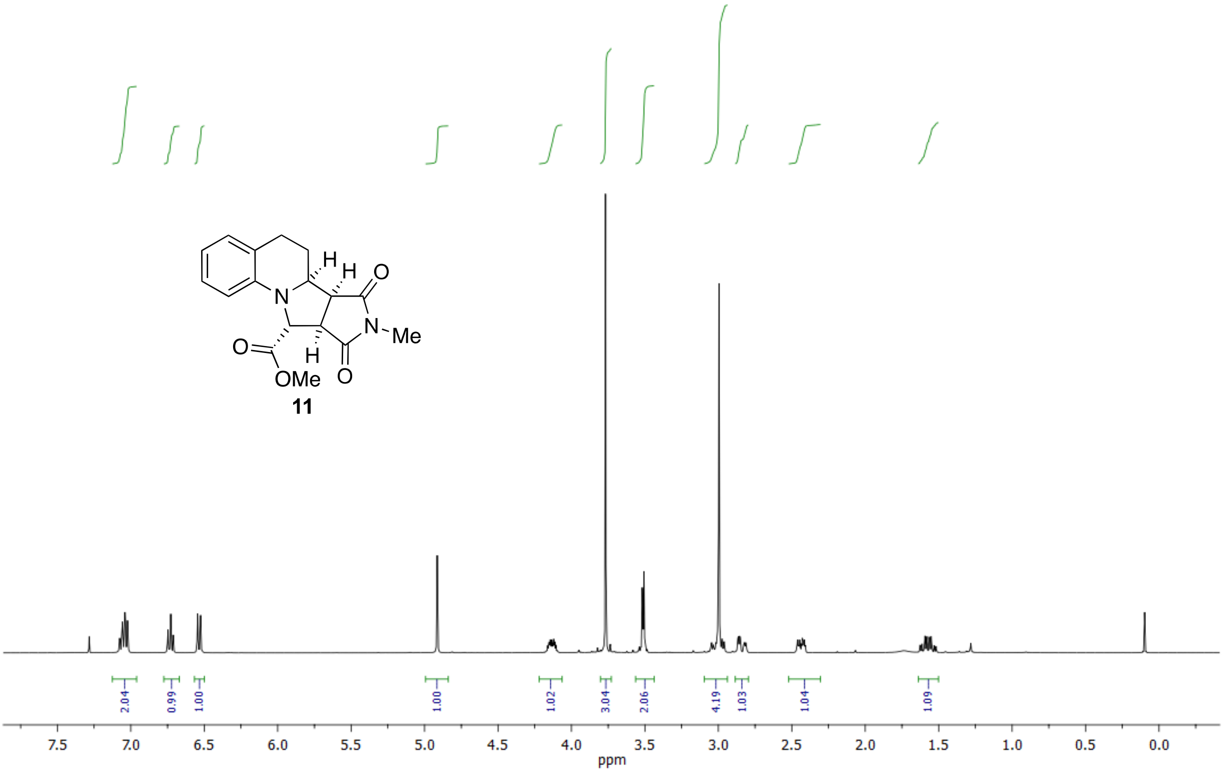
H
H
O
N
N
Me
O
H
OMe
O
11
2.04
0.99
1.00
1.00
1.02
3.04
2.06
4.19
1.03
1.04
1.09
7.5
7.0
6.5
6.0
5.5
5.0
4.5
4.0
3.5
3.0
2.5
2.0
1.5
1.0
0.5
0.0
ppm

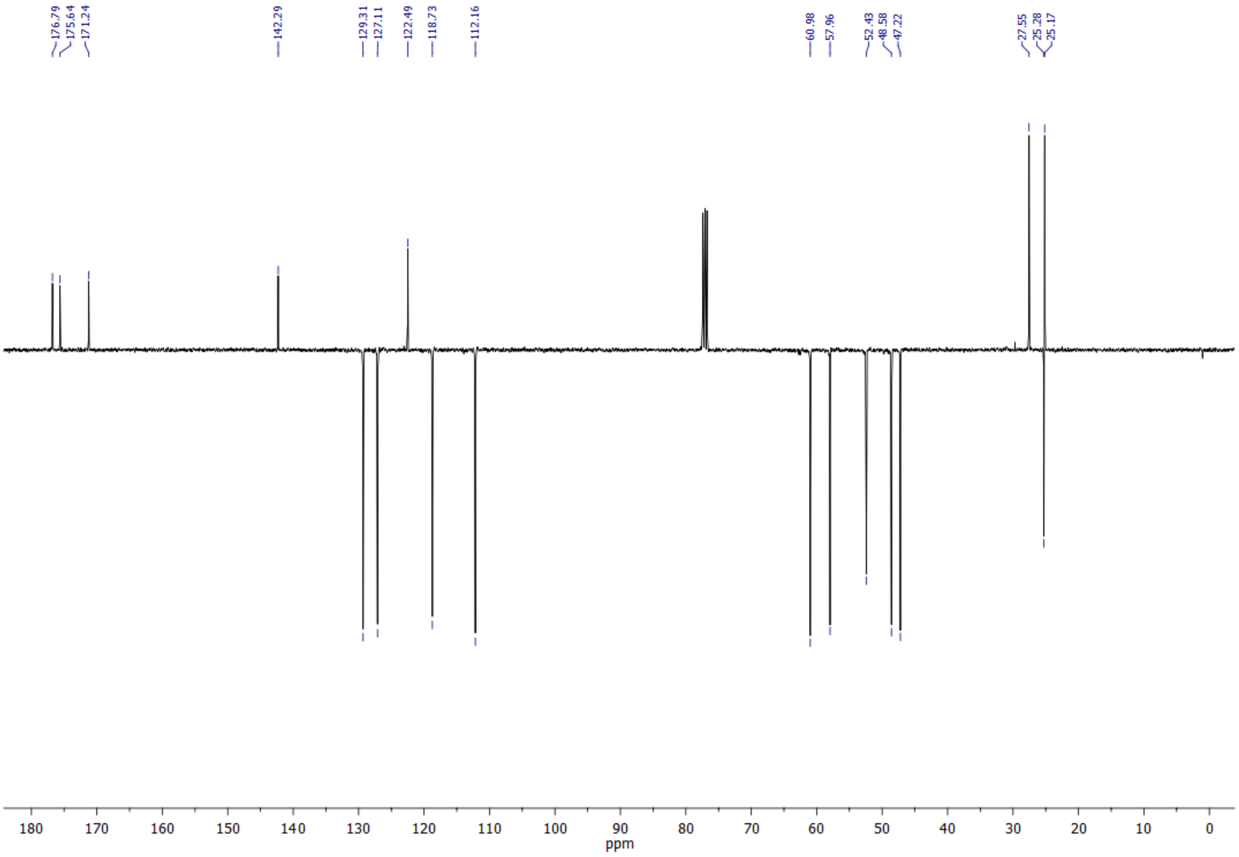
176.79
175.64
171.24
142.29
129.31
127.11
122.49
118.73
112.16
60.98
57.96
52.43
48.58
47.22
27.55
25.28
25.17
180
170
160
150
140
130
120
110
100
90
80
70
60
50
40
30
20
10
0
ppm

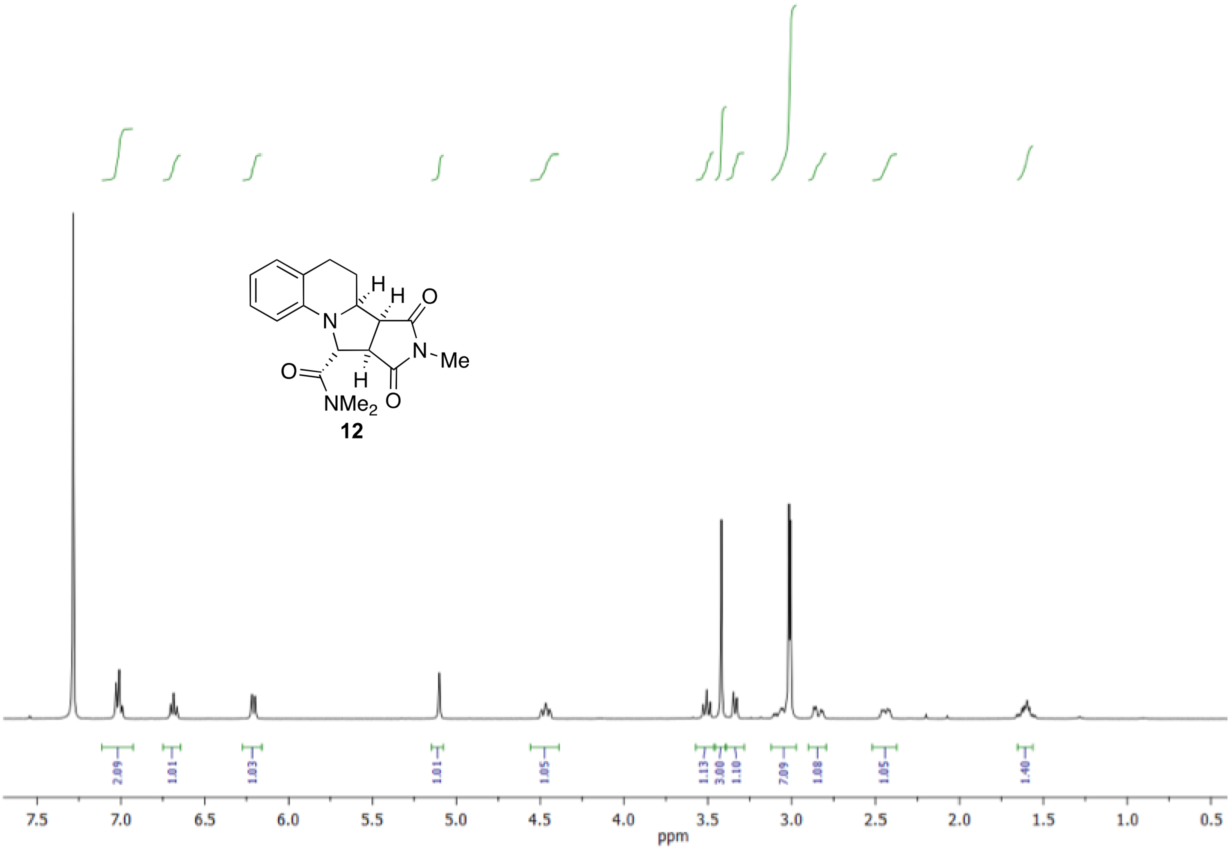
H
H
O
N
Me
O
NMe$_2$
O
H
12
2.09
1.01
1.03
1.01
1.05
1.13
3.00
1.10
7.09
1.08
1.05
1.40
7.5 7.0 6.5 6.0 5.5 5.0 4.5 4.0 3.5 3.0 2.5 2.0 1.5 1.0 0.5
ppm

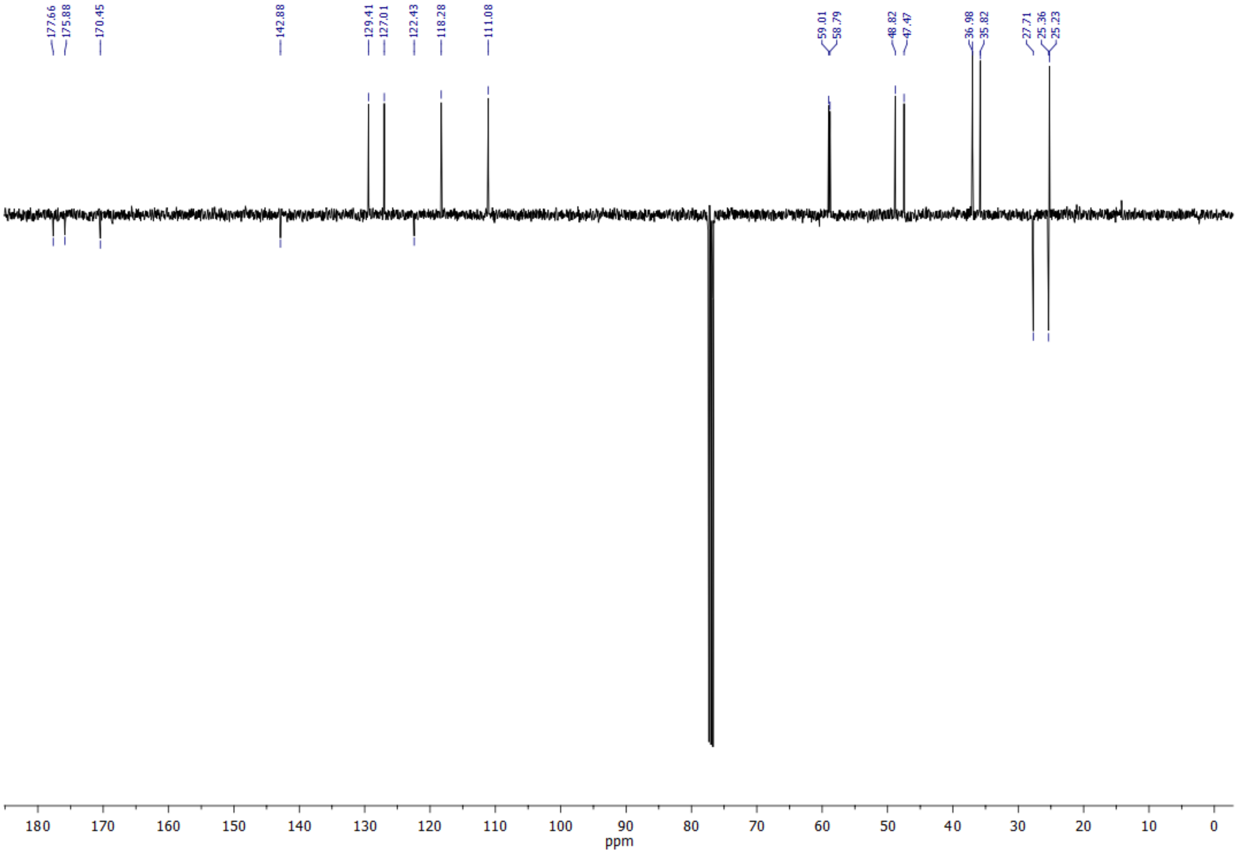
177.66
175.88
170.45
142.88
129.41
127.01
122.43
118.28
111.08
59.01
58.79
48.82
47.47
36.98
35.82
27.71
25.36
25.23
180 170 160 150 140 130 120 110 100 90 80 70 60 50 40 30 20 10 0
ppm

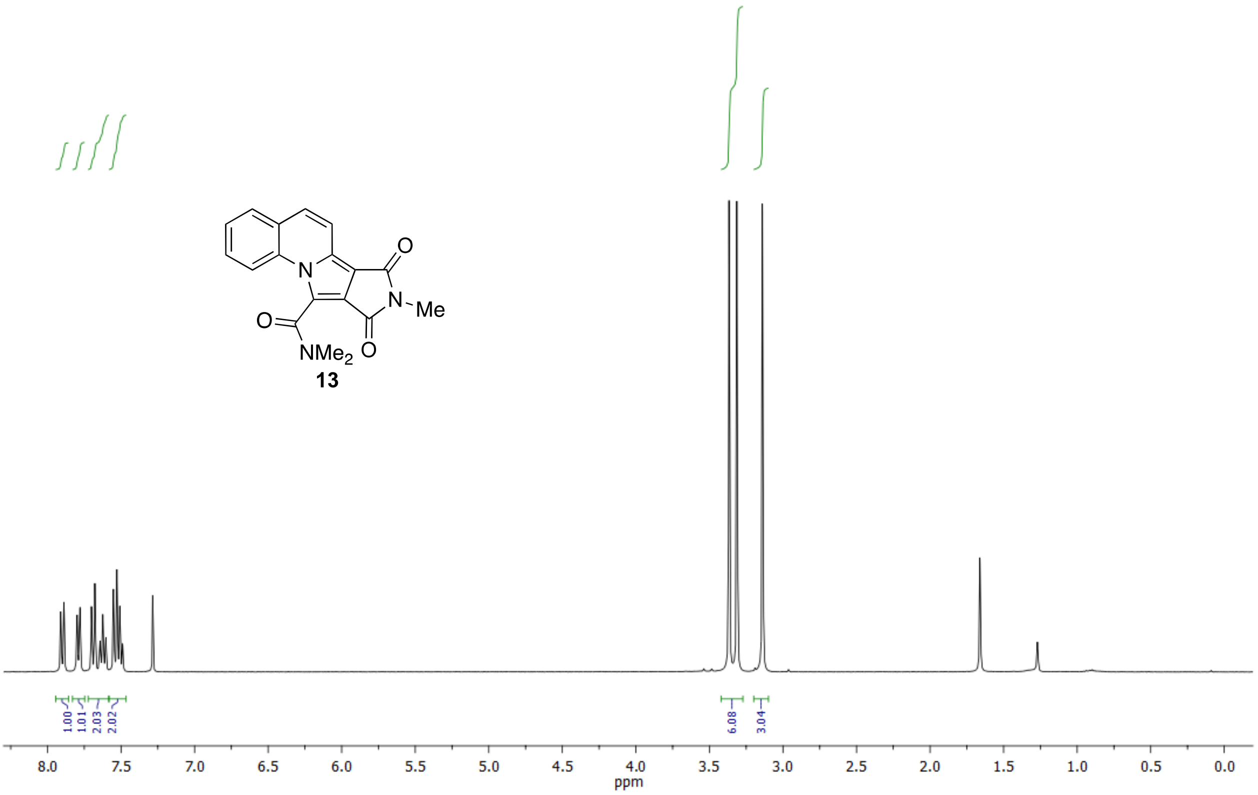

O
N
Me
NMe2
13
1.00
1.01
2.03
2.02
6.08
3.04
8.0
7.5
7.0
6.5
6.0
5.5
5.0
4.5
4.0
ppm
3.5
3.0
2.5
2.0
1.5
1.0
0.5
0.0

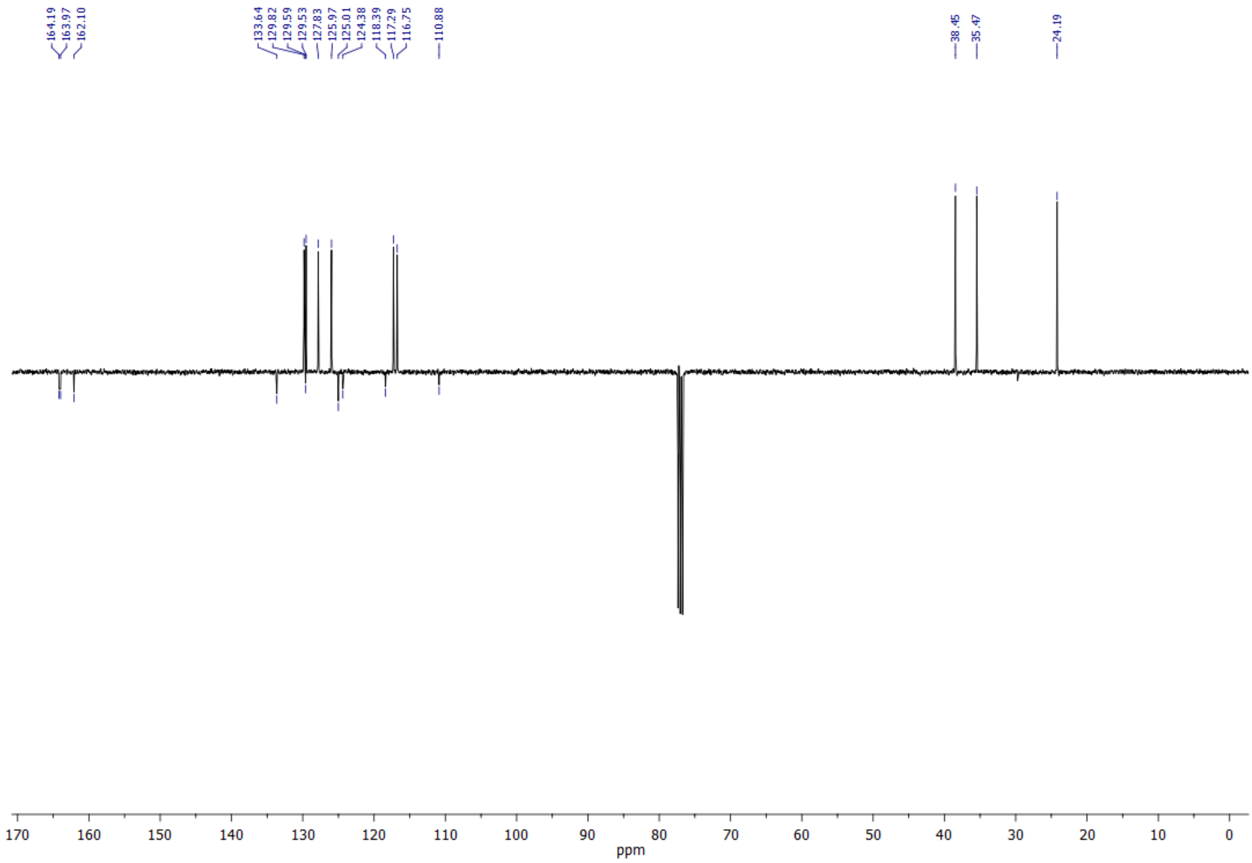

164.19
163.97
162.10
133.64
129.82
129.59
129.53
127.83
125.97
125.01
124.38
118.39
117.29
116.75
110.88
38.45
35.47
24.19
170
160
150
140
130
120
110
100
90
80
ppm
70
60
50
40
30
20
10
0

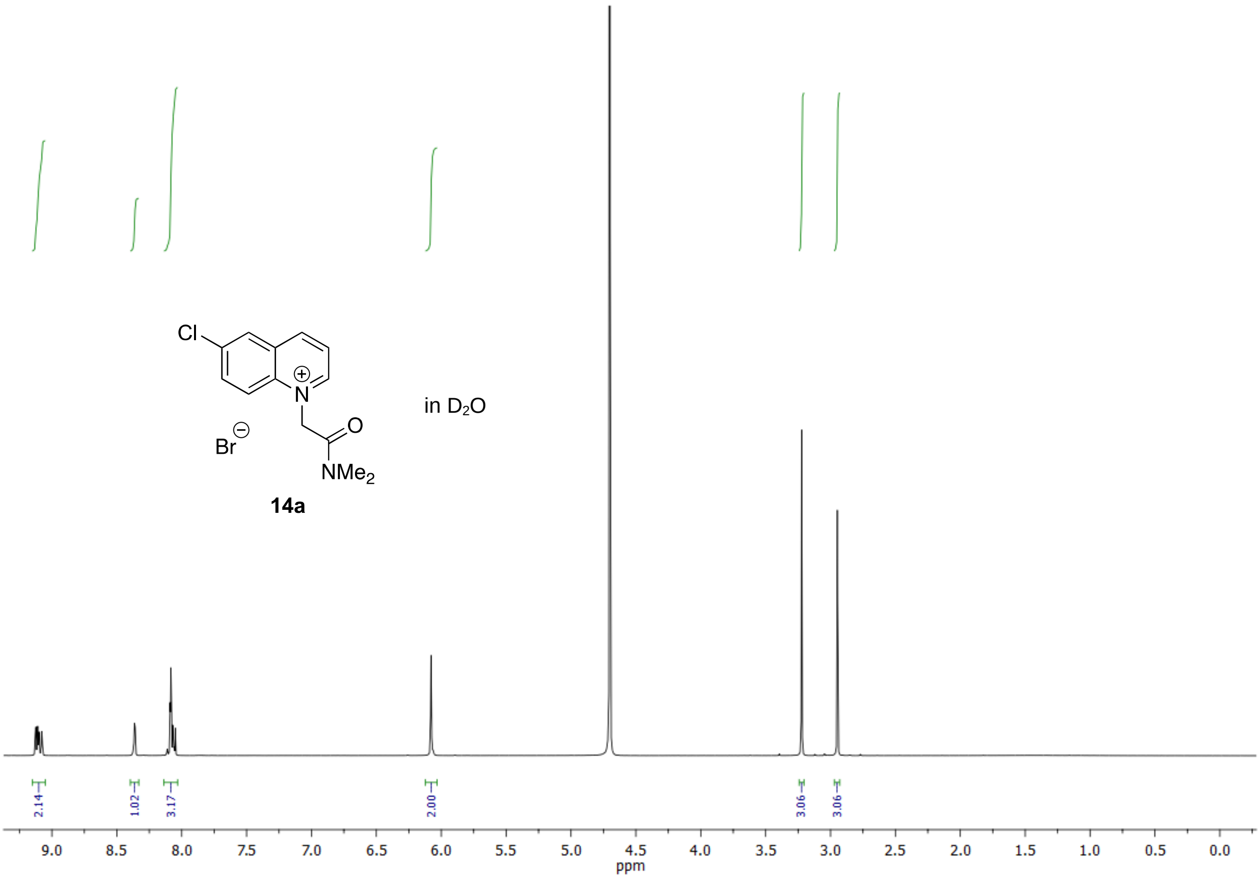
Cl
N
O
Br
NMe2
14a
in D2O
2.14
1.02
3.17
2.00
3.06
3.06
9.0 8.5 8.0 7.5 7.0 6.5 6.0 5.5 5.0 4.5 4.0 3.5 3.0 2.5 2.0 1.5 1.0 0.5 0.0
ppm

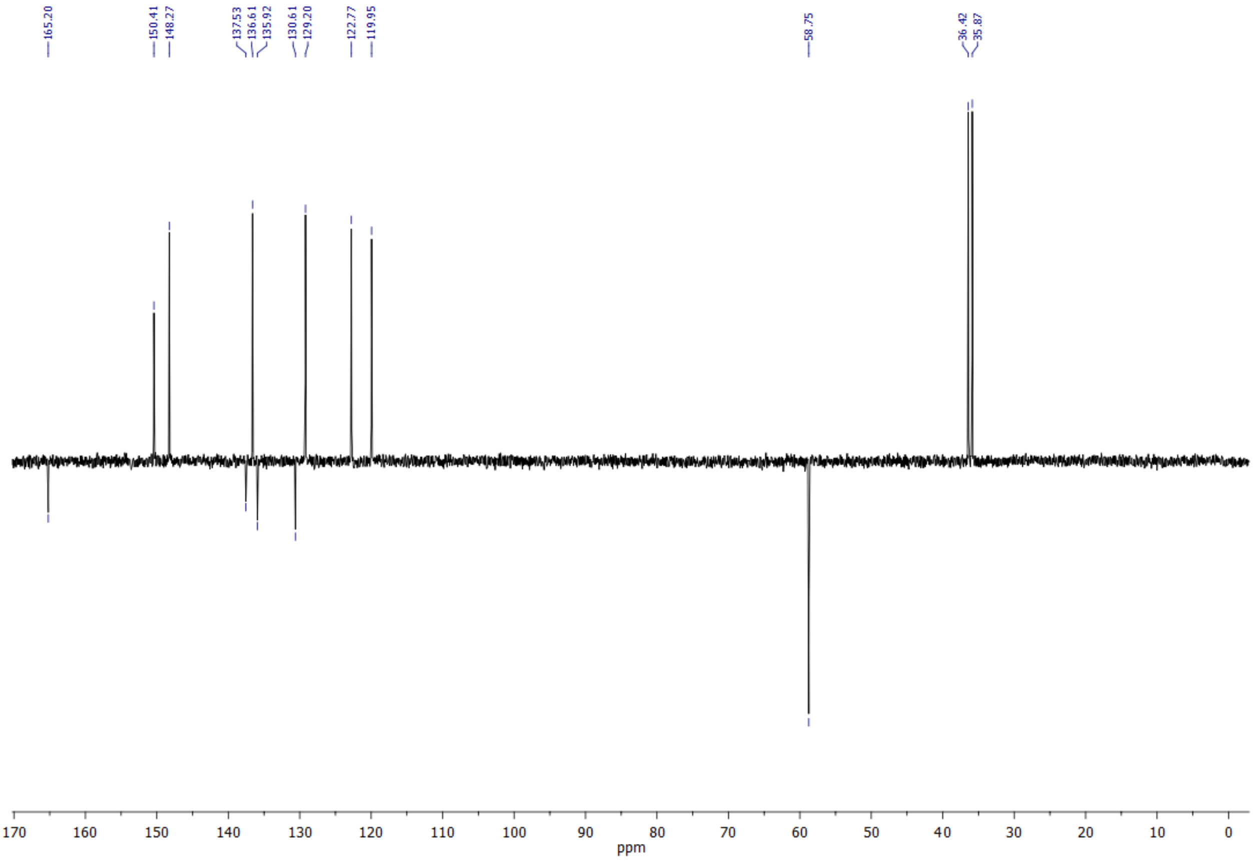
165.20
150.41
148.27
137.53
136.61
135.92
130.61
129.20
122.77
119.95
58.75
36.42
35.87
170 160 150 140 130 120 110 100 90 80 70 60 50 40 30 20 10 0
ppm

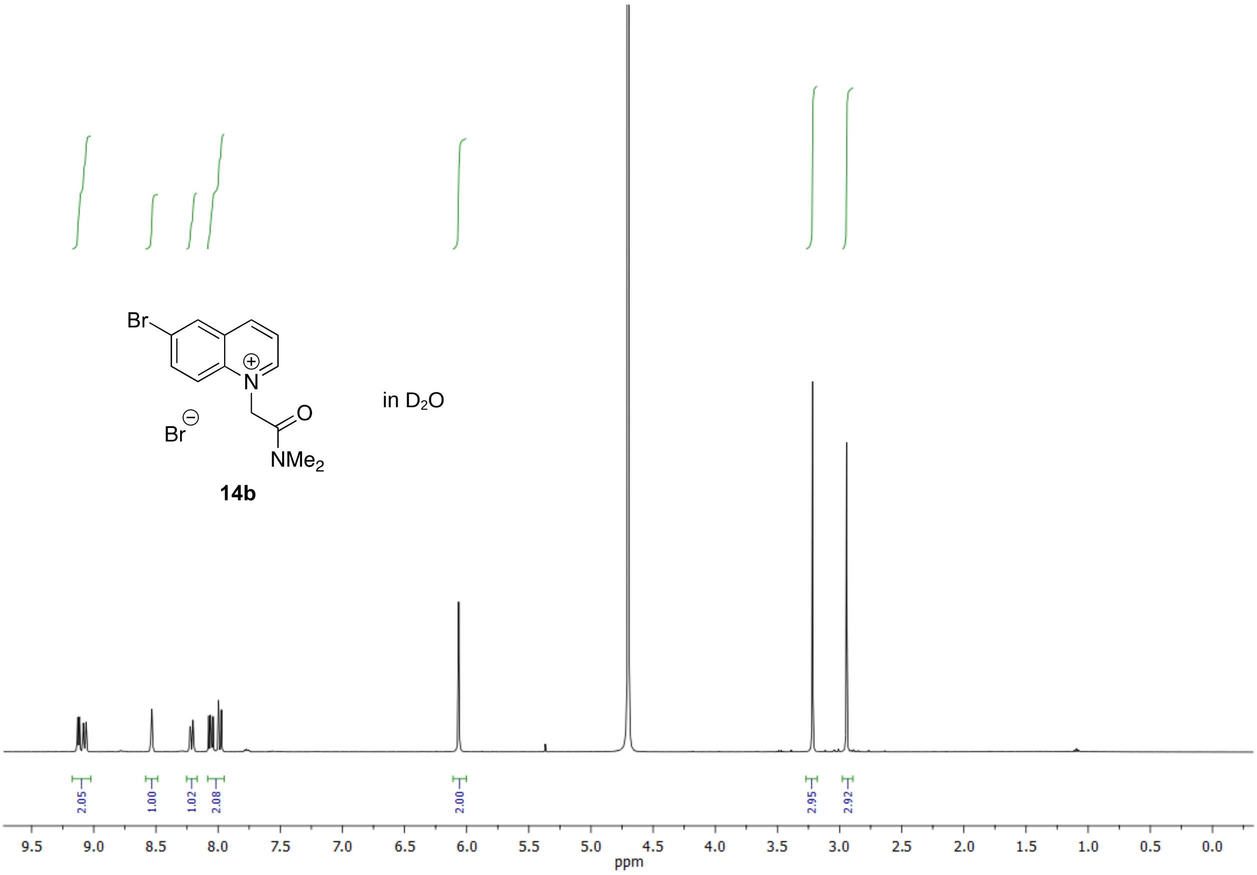
Br
N
O
Br
NMe$_2$
2.05
1.00
1.02
2.08
2.00
2.95
2.92
9.5 9.0 8.5 8.0 7.5 7.0 6.5 6.0 5.5 5.0 4.5 4.0 3.5 3.0 2.5 2.0 1.5 1.0 0.5 0.0
ppm

14b

in D_2O

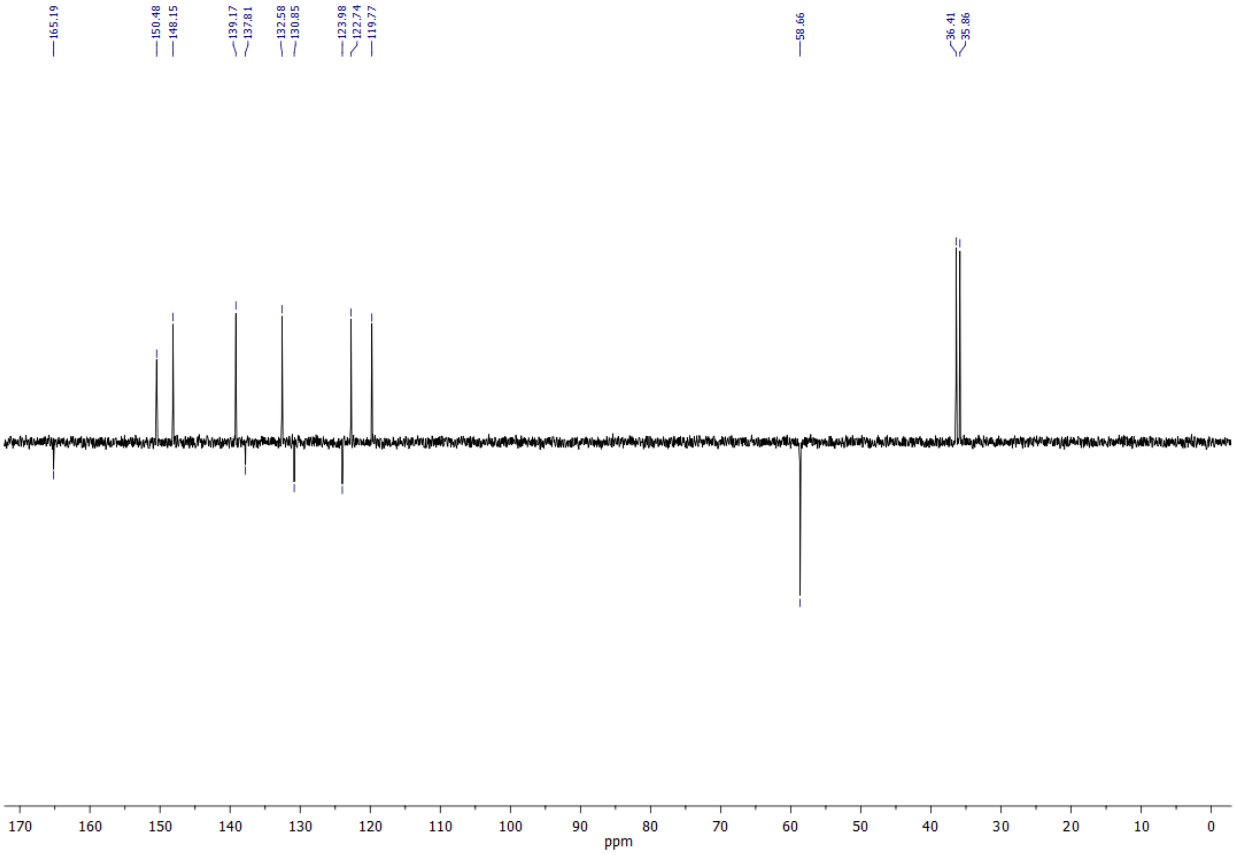
165.19
150.48
148.15
139.17
137.81
132.58
130.85
123.98
122.74
119.77
58.66
36.41
35.86
170 160 150 140 130 120 110 100 90 80 70 60 50 40 30 20 10 0
ppm

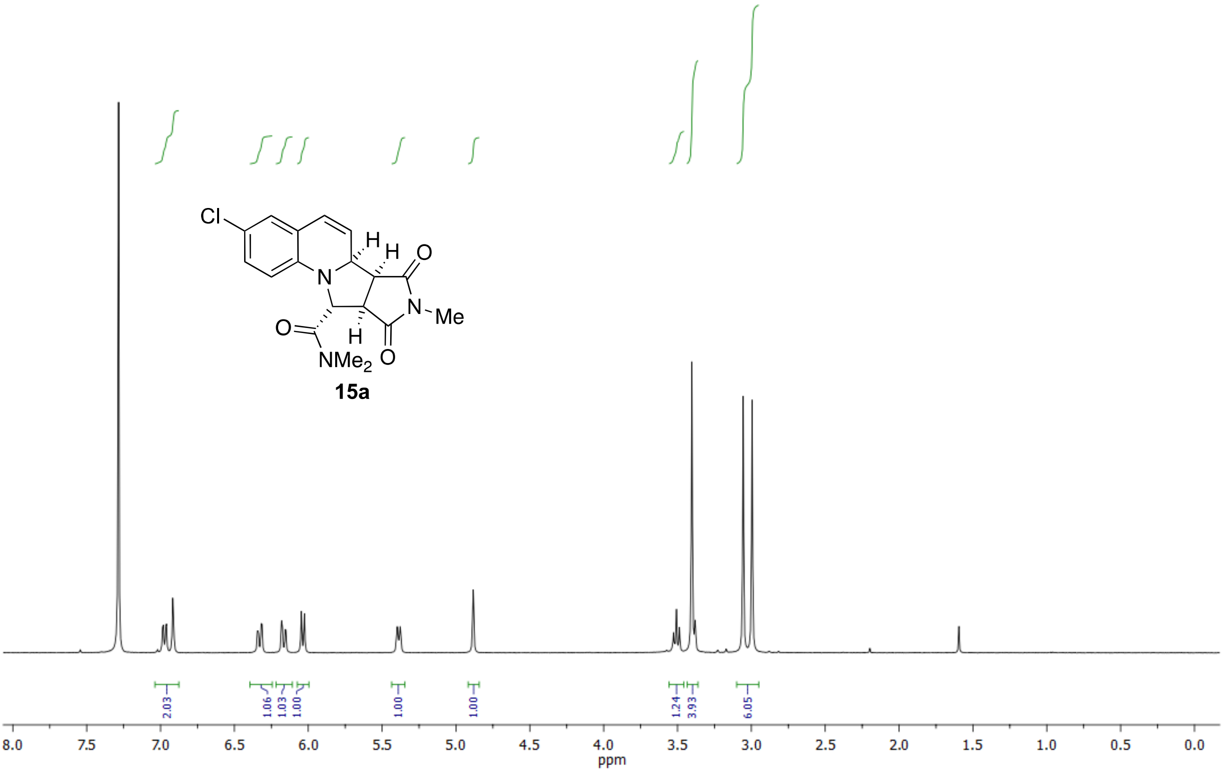
Cl
H
H
O
N
Me
O
NMe2
H
O
15a
2.03
1.06
1.03
1.00
1.00
1.00
1.24
3.93
6.05
8.0 7.5 7.0 6.5 6.0 5.5 5.0 4.5 4.0 3.5 3.0 2.5 2.0 1.5 1.0 0.5 0.0
ppm

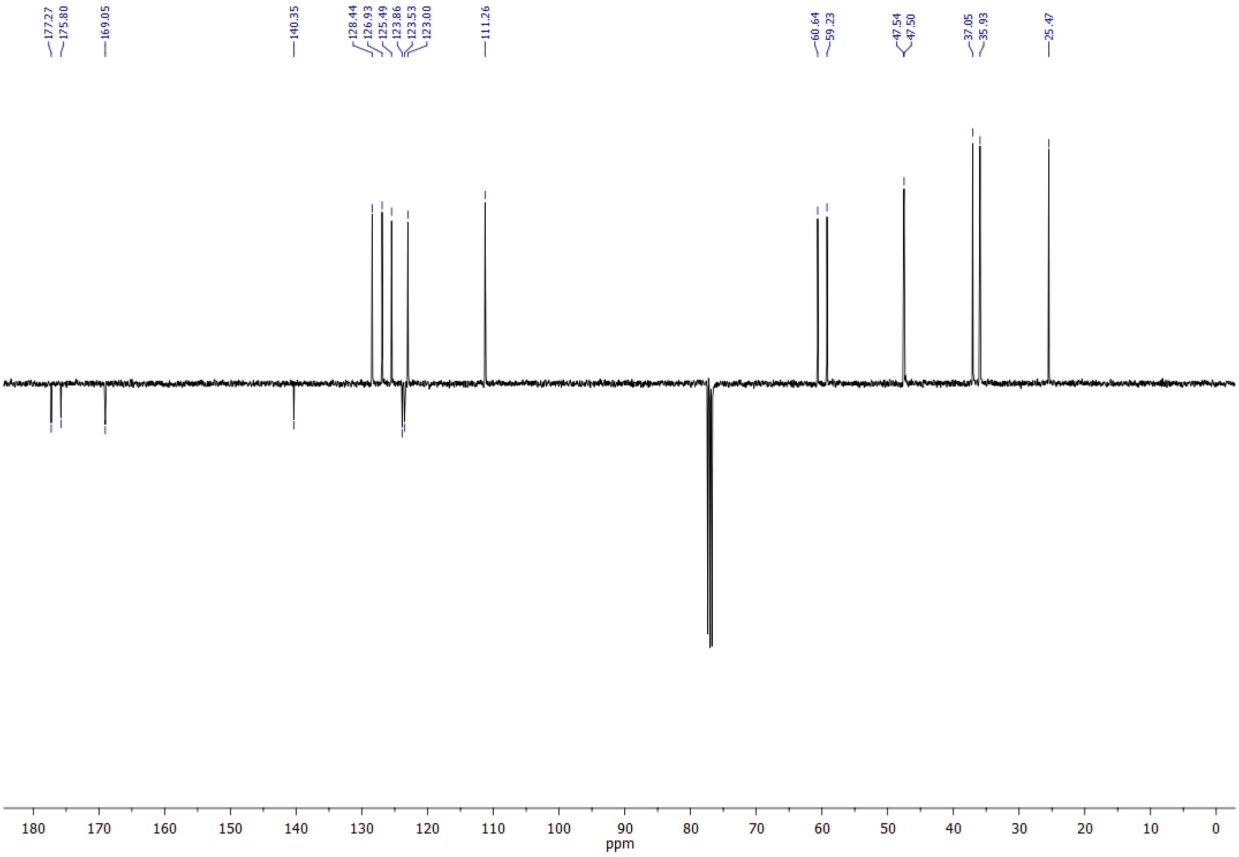
177.27
175.80
169.05
140.35
128.44
126.93
125.49
123.86
123.53
123.00
111.26
60.64
59.23
47.54
47.50
37.05
35.93
25.47
180 170 160 150 140 130 120 110 100 90 80 70 60 50 40 30 20 10 0
ppm

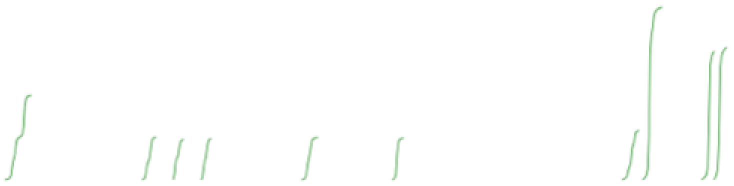

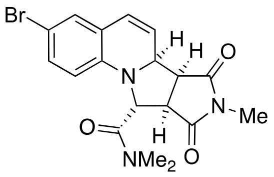

Br
H
H
O
N
N
Me
O
H
NMe2
O

15b

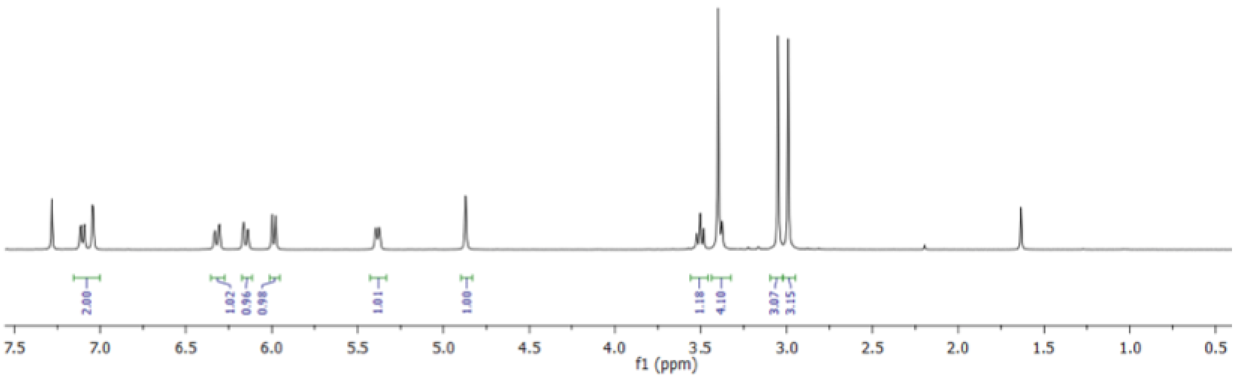

2.00
1.02
0.96
0.98
1.01
1.00
1.18
4.10
3.07
3.15
7.5
7.0
6.5
6.0
5.5
5.0
4.5
4.0
3.5
3.0
2.5
2.0
1.5
1.0
0.5
f1 (ppm)

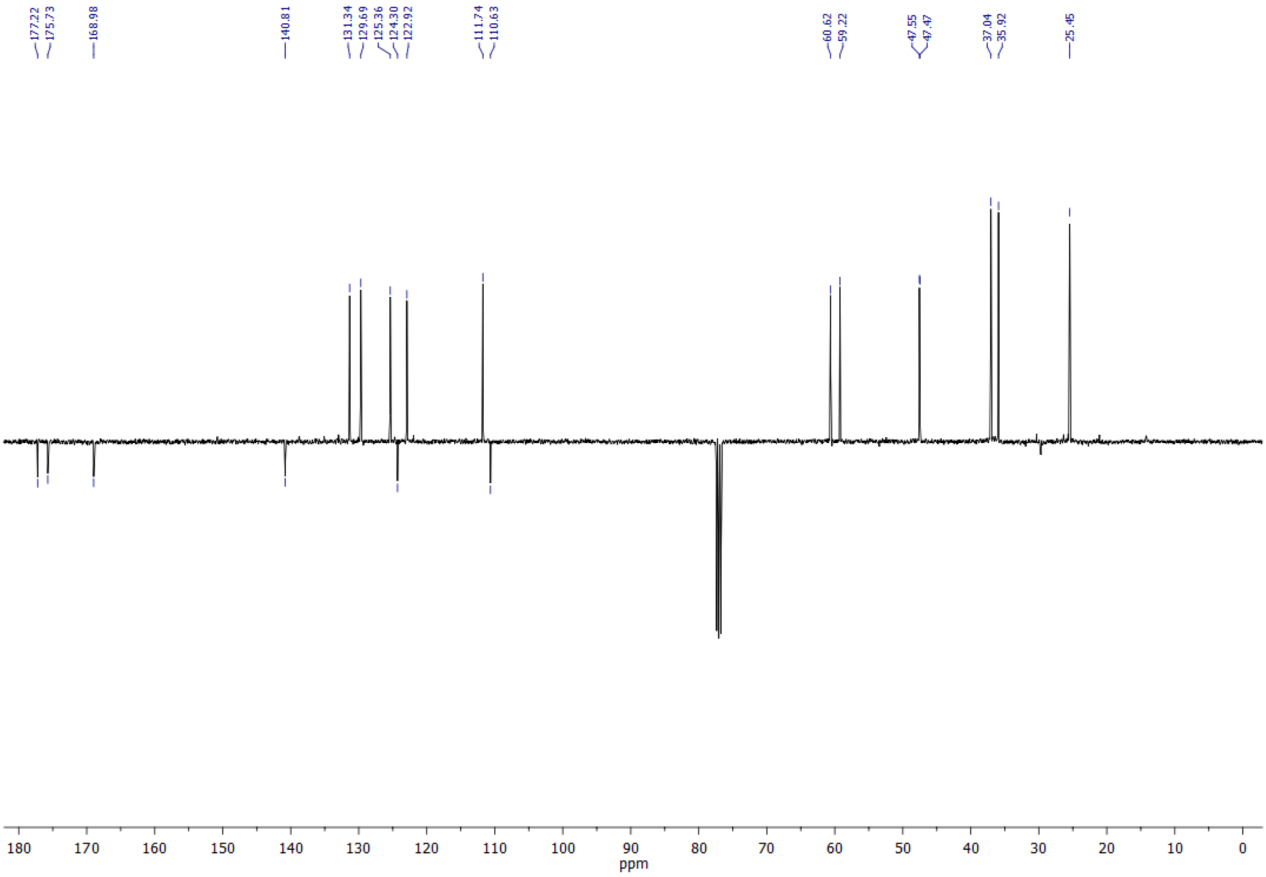

177.22
175.73
168.98
140.81
131.34
129.69
125.36
124.30
122.92
111.74
110.63
60.62
59.22
47.55
47.47
37.04
35.92
25.45
180
170
160
150
140
130
120
110
100
90
80
70
60
50
40
30
20
10
0
ppm

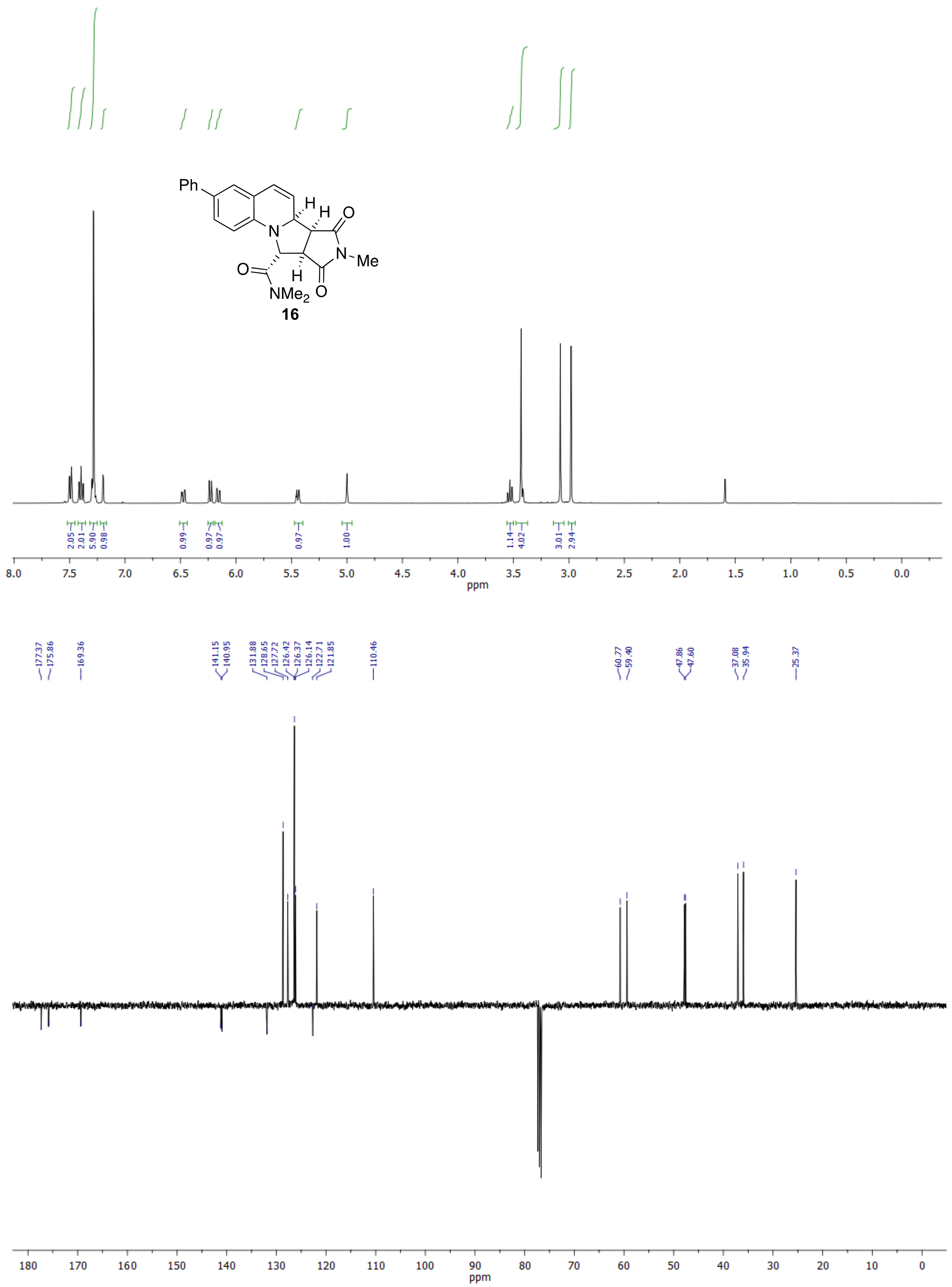

Supplement: File 1 — Experimental procedures, spectroscopic and X-ray data (CCDC 1907018–1907020 for compounds 7c, 9 and 15a) and copies of NMR spectra. [file Beilstein_J_Org_Chem-15-1480-s001.pdf]
